# Root angle is controlled by *EGT1* in cereal crops employing an antigravitropic mechanism

**DOI:** 10.1073/pnas.2201350119

**Published:** 2022-07-26

**Authors:** Riccardo Fusi, Serena Rosignoli, Haoyu Lou, Giuseppe Sangiorgi, Riccardo Bovina, Jacob K. Pattem, Aditi N. Borkar, Marco Lombardi, Cristian Forestan, Sara G. Milner, Jayne L. Davis, Aneesh Lale, Gwendolyn K. Kirschner, Ranjan Swarup, Alberto Tassinari, Bipin K. Pandey, Larry M. York, Brian S. Atkinson, Craig J. Sturrock, Sacha J. Mooney, Frank Hochholdinger, Matthew R. Tucker, Axel Himmelbach, Nils Stein, Martin Mascher, Kerstin A. Nagel, Laura De Gara, James Simmonds, Cristobal Uauy, Roberto Tuberosa, Jonathan P. Lynch, Gleb E. Yakubov, Malcolm J. Bennett, Rahul Bhosale, Silvio Salvi

**Affiliations:** ^a^School of Biosciences, University of Nottingham, LE12 5RD Nottingham, United Kingdom;; ^b^Future Food Beacon of Excellence, University of Nottingham, LE12 5RD Nottingham, United Kingdom;; ^c^Department of Agricultural and Food Sciences, University of Bologna, 40127 Bologna, Italy;; ^d^School of Agriculture, Food and Wine, Waite Research Institute, University of Adelaide, Urrbrae, SA 5064, Australia;; ^e^Australian Research Council Centre of Excellence in Plant Cell Walls, University of Adelaide, Glen Osmond, SA 5062, Australia;; ^f^Food Sciences, University of Nottingham, LE12 5RD Nottingham, United Kingdom;; ^g^National Centre for Molecular Hydrodynamics, and Soft Matter Biomaterials and Bio-interfaces, University of Nottingham, LE12 5RD, United Kingdom;; ^h^School of Veterinary Medicine and Science, University of Nottingham, LE12 5RD Nottingham, United Kingdom;; ^i^Wolfson Center for Global Virus Research, University of Nottingham, LE12 5RD Nottingham, United Kingdom;; ^j^Department of Science and Technology for Humans and the Environment, Università Campus Bio-Medico di Roma, Rome, 00128 Italy;; ^k^Institute of Crop Sciences and Resource Conservation, Crop Functional Genomics, University of Bonn, 53113 Bonn, Germany;; ^l^Biosciences Division, Oak Ridge National Laboratory, Oak Ridge, TN 37830;; ^m^Leibniz Institute of Plant Genetics and Crop Plant Research, 06466 Gaterslelben, Germany;; ^n^Center of integrated Breeding Research, Department of Crop Sciences, Georg-August-University, 37075 Göttingen, Germany;; ^o^German Centre for Integrative Biodiversity Research Halle-Jena-Leipzig, 04103 Leipzig, Germany;; ^p^Institute of Bio- and Geo-sciences, Plant Sciences, 52428 Juelich, Germany;; ^q^John Innes Centre, Norwich Research Park, NR4 7UH Norwich, United Kingdom;; ^r^Department of Plant Science, Pennsylvania State University, University Park, PA 16802

**Keywords:** root angle, antigravitropic, cell-wall, barley, wheat

## Abstract

Root angle in crops represents a key trait for efficient capture of soil resources. Root angle is determined by competing gravitropic versus antigravitropic offset (AGO) mechanisms. Here we report a root angle regulatory gene termed *ENHANCED GRAVITROPISM1* (*EGT1*) that encodes a putative AGO component, whose loss-of-function enhances root gravitropism. Mutations in barley and wheat *EGT1* genes confer a striking root phenotype, where every root class adopts a steeper growth angle. *EGT1* encodes an F-box and Tubby domain-containing protein that is highly conserved across plant species. Haplotype analysis found that natural allelic variation at the barley *EGT1* locus impacts root angle. Gravitropic assays indicated that *Hvegt1* roots bend more rapidly than wild-type. Transcript profiling revealed *Hvegt1* roots deregulate reactive oxygen species (ROS) homeostasis and cell wall-loosening enzymes and cofactors. ROS imaging shows that *Hvegt1* root basal meristem and elongation zone tissues have reduced levels. Atomic force microscopy measurements detected elongating *Hvegt1* root cortical cell walls are significantly less stiff than wild-type. In situ analysis identified *HvEGT1* is expressed in elongating cortical and stele tissues, which are distinct from known root gravitropic perception and response tissues in the columella and epidermis, respectively. We propose that EGT1 controls root angle by regulating cell wall stiffness in elongating root cortical tissue, counteracting the gravitropic machinery’s known ability to bend the root via its outermost tissues. We conclude that root angle is controlled by *EGT1* in cereal crops employing an antigravitropic mechanism.

## Significance

The growth angle roots adopt are critical for capturing soil resources, such as nutrients and water. Despite its agronomic importance, few regulatory genes have been identified in crops. Here we identify the root angle regulatory gene *ENHANCED GRAVITROPISM 1* (*EGT1*) in barley. Strikingly, mutants lacking *EGT1* exhibit a steeper angle in every root class. EGT1 appears to function as a component of an antigravitropic offset mechanism that regulates tissue stiffness, which impacts final root growth angle. *EGT1* is a hot spot for selection as natural allelic variation within a conserved Tubby domain that is linked with steeper root angle. Analogous *EGT1*-dependent regulation of root angle in wheat demonstrates broad significance of EGT1 for trait improvement in cereal crops.

Root architectural traits, such as angle, play a critical role in adapting to different environmental conditions and capturing soil resources, such as water and nutrients. For example, steeper root growth angle is advantageous for accessing subsoil water and enhancing drought tolerance and improving nitrogen (N) capture, while shallow root growth angle improves capture of phosphorus (P) from topsoil (1–3). Moreover, recent studies report that modified root angle increases yield under saline conditions (4). Thus, improved understanding of the genes and mechanisms controlling root growth angle would facilitate breeding of crop varieties better suited for different abiotic stresses arising from future climatic conditions.

The growth angles of different root classes (e.g., primary, seminal, lateral, and crown) are often distinct to limit competition. These distinct angles are referred to as gravitropic set-point angle (GSA). The GSA of different root classes is determined by competing gravitropic and antigravitropic offset (AGO) mechanisms (5, 6). The gravitropic mechanism has been extensively studied in *Arabidopsis thaliana* roots. These studies have identified that change in root orientation is perceived in columella cells at the root tip, triggering formation of a lateral auxin gradient that root cap cells transport to epidermal cells in the elongation zone, leading to differential root growth and bending (7–9). In contrast to the detailed knowledge about the genes, signals and mechanisms involved in the root gravitropic response, the AGO mechanism has only recently begun to be unraveled (5, 6). Auxin transport has also been linked with the AGO mechanism, implying that the interaction of two opposing gravitropic and AGO-regulated auxin fluxes could determine the angle of organ growth (5, 10). However, detailed knowledge about auxin-dependent or auxin-independent components of AGO mechanisms still remains unclear.

Here, we report a putative component of the AGO mechanism in cereal roots termed *ENHANCED GRAVITROPISM 1* (*EGT1*). Screening of a barley TILLING mutant collection identified a mutant exhibiting a striking steep root growth angle phenotype. Bulk segregant analysis (BSA) mapped the mutation within a 130-Mb region on chromosome 6. Exome and whole-genome shotgun sequencing (WGS) identified mutations in the coding sequence of *HORVU6Hr1G068970* (Tubby-like F-box protein). TILLING studies revealed EGT1 function is also conserved in durum wheat. *HvEGT1* is highly expressed in root stele tissues distinct from known auxin-mediated gravity-responsive root cap and epidermal tissues. *HvEGT1* appears to function in an auxin-independent AGO mechanism. RNA sequencing (RNA-seq) revealed many peroxidases and cell wall softening/stiffening enzymes are differentially regulated in *hvegt1* mutant root tips compared to wild-type. Atomic force microscopy (AFM) measurements revealed elongation zone cell walls of *Hvegt1* roots are significantly less stiff than wild-type. We propose that *HvEGT1* controls root growth angle by functioning as an AGO component in an auxin-independent pathway in elongating root tissues via regulation of cell wall stiffening and loosening, thereby serving to counteract gravitropic bending in the outermost tissues.

## Results

### Barley Mutant TM194 Exhibits Steeper Root Growth Angle in Every Root Class.

**Fig. 1.**
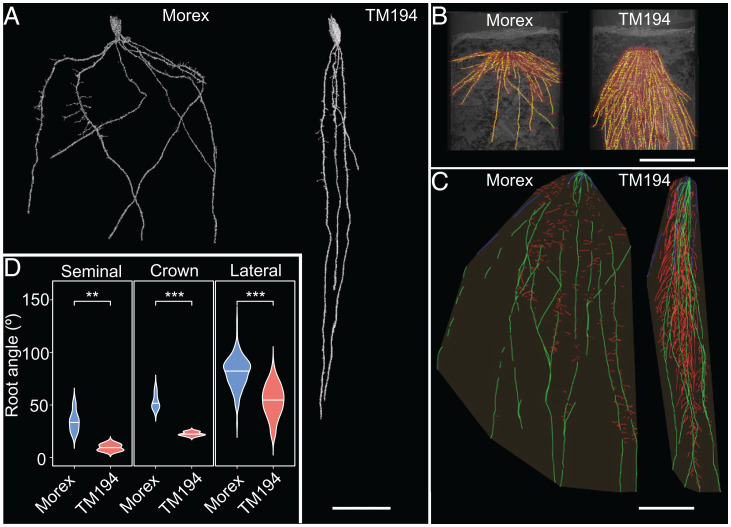
TM194 mutant shows steeper angle in every root class in soil conditions. (*A*) Representative X-ray microCT scan image of 10 DAG wild-type (Morex) and TM194 roots, showing major differences in seminal root vertical angle. (Scale bar, 2 cm.) (*B*) Representative X-ray CT scan image of fully grown plants at grain maturation stage revealing major difference in crown root vertical angle between Morex and TM194. (Scale bar, 10 cm.) (*C*) Representative image of 20 DAG Morex and TM194 revealing difference in lateral root insertion angles (red colored). (Scale bar, 10 cm.) (*D*) Quantification of vertical root angle from segmented seminal roots, crown roots, and lateral roots. Asterisks indicate statistically significant difference using Welch’s *t* test at ***P < 0.001 and **P < 0.001 in *n* > 4 independent replicates, respectively.

A barley root mutant line, TM194, exhibiting a striking steeper seminal and lateral root phenotype (*SI Appendix*, Fig. S1) was initially identified in a chemically mutagenized population of the cv. Morex (11) using a semihydroponic rhizotron screening system. Three-dimensional (3D) root architecture phenotyping of 10-d-old TM194 roots using X-ray microcomputed tomography (microCT) (12) revealed the steeper seminal root angle phenotype directly in soil (Fig. 1*A*). Phenotyping TM194 roots 20 d after germination (DAG, using soil-filled rhizotrons) and at grain maturation stage (using X-ray CT) revealed lateral and crown root angles are also significantly steeper compared to wild-type Morex (Fig. 1 *B*–*D*). Hence, the TM194 mutant exhibited steeper root growth angle in every root class examined, in both semihydroponic and soil conditions. In contrast, no significant difference in shoot growth angle (*P* value 0.4819) (*SI Appendix*, Fig. S2*A*) at seedling stage or leaf growth angle (*P* value 0.566) (*SI Appendix*, Fig. S2*B*) at the flowering stage was observed in the TM194 mutant compared to wild-type. Hence, the TM194 mutation causes a root-specific angle defect.

### TM194 Root Angle Defect Is Caused by a Mutation in *ENHANCED GRAVITROPISM 1* (*HvEGT1*).

**Fig. 2.**
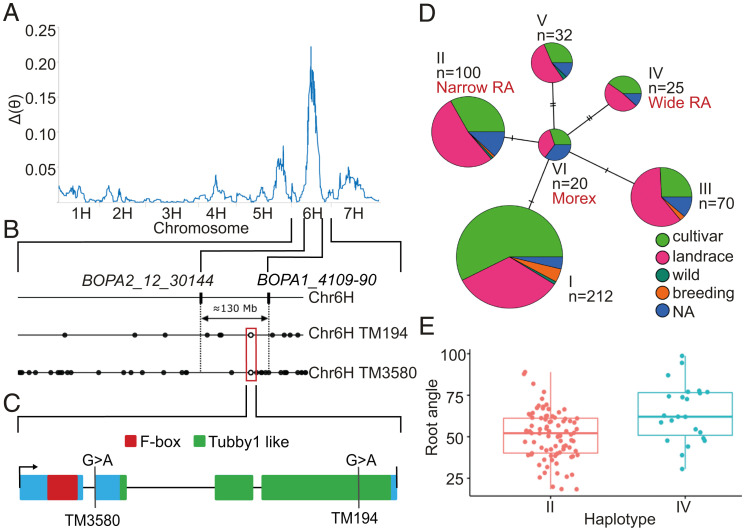
Exome sequencing of TM194 and WGS of TM194 and TM3580 identify mutations in *EGT1.* (*A* and *B*) SNP-based BSA from F2 plants from TM194 x cv. Barke outcross. (*A*) Genome-wide plot of unbalanced allelic frequency from SNP-based BSA. The Δθ parameter represents the difference in allele frequency for each tested SNP. (*B*) Schematic representing a region spanning ∼c.130 Mb on chromosome 6H between markers *BOPA2_12_30144* and *BOPA1_4109-90*. Filled circles indicate all SNPs within genes present in this region while empty circles (in red rectangle) indicate SNPs within pinpointed *HORVU6Hr1G068970* gene. WGS of another mutant allele of *HORVU6Hr1G068970* also showed steeper root growth angle phenotype. This gene is further named as *EGT1*. (*C*) Schematic representation of *EGT1* and the position of the two mutations in relation to the F-box domain (red) and the Tubby-like protein domains. (*D* and *E*) Haplotype analysis of *EGT1* nucleotide sequence variation present in WHEALBI barley germplasm collection. (*D*) Haplotype network analysis revealed that haplotype II and IV carry missense substitutions, while the remaining four haplotypes carry synonymous substitutions. *n* indicates number of genotypes within each class. (*E*) Root growth angle distribution of WHEALBI barley lines carrying haplotype II (86 lines) and IV (25 lines).

To discover the genetic and molecular basis of the TM194 root growth angle phenotype, the mutant was initially outcrossed to Barke, a distinct barley variety that exhibits a similar root growth angle phenotype to cv. Morex. While F1 plants exhibited a wild-type phenotype, F2 plants (*n* = 75) segregated in a Mendelian pattern for either a steeper or wild-type seminal root phenotype (59:16 plants, wild-type vs. steeper, respectively, χ^2^ 3:1, n.s.), consistent with the TM194 root growth angle phenotype segregating as a single recessive allele. Using the same F2 population, a single nucleotide polymorphism (SNP)-based BSA revealed that the mutated locus mapped to chromosome 6 (Fig. 2*A*) in a large pericentromeric region spanning ∼130 Mb between markers *BOPA2_12_30144* and *BOPA1_4109-90*.

To pinpoint the root angle mutation, exome and whole genome sequencing was performed on TM194. This revealed missense mutations in four genes within the chromosome 6H region highlighted by BSA (Fig. 2 *B* and *C* and Dataset S1). To pinpoint the relevant gene, we whole genome sequenced a second independent root angle mutant allele, termed TM3580 (*SI Appendix*, Fig. S3). TM3580 contained six mutations in the same chromosome region, while only one mutation coincided with TM194 in an overlapping gene *HORVU6Hr1G068970* (encoding Tubby-like F-box protein) (Dataset S1, highlighted in red). F1 progenies of a genetic cross between TM3580 and TM194 did not complement steeper root growth angle phenotype (*SI Appendix*, Fig. S4), confirming that these mutants are allelic at locus *HORVU6Hr1G068970*. Specifically, TM3580 contained a mutation in the first intron of *HORVU6Hr1G068970*, predicted to cause a splice acceptor variant (Dataset S1). Bulk RNA-seq analysis of TM3580 and Morex root samples confirmed the TM3580 mutation caused a splice acceptor variant, resulting in a deletion of nine amino acids without any frameshift (*SI Appendix*, Fig. S5). Interestingly, neither mutation significantly affects *HORVU6Hr1G068970* expression level (*SI Appendix*, Fig. S6), suggesting their steeper root phenotype is due to altered HvEGT1 protein structure or function. Taken together, these results provide conclusive evidence that mutations in *HORVU6Hr1G068970* are responsible for the steeper root angle phenotype, leading us to name this gene as barley *ENHANCED GRAVITROPISM 1* (*HvEGT1*).

### Mutations in *HvEGT1* Tubby Domain Disrupt Gene Function.

Next, we examined whether nucleotide polymorphisms within *HvEGT1* could provide a source of natural variation in root growth angle observed in barley diversity panels. We exploited the availability of exome sequence of a large barley germplasm collection (WHEALBI collection) (13). Using haplotype network analysis of nucleotide sequence variation within the *HvEGT1* coding sequence, we identified two haplotypes (II and IV) carrying missense substitutions and four other haplotypes carrying synonymous substitutions (I, III, V, and VI) (Fig. 2*D*). Based on this result, we phenotyped barley accessions carrying haplotypes II (*n* = 86) and IV (*n* = 25) using a semihydroponic system. Accessions carrying haplotype II exhibited significantly steeper seminal root angle distribution than accessions carrying haplotype IV (50.9 ± 14.8 vs. 64.3 ± 17.6, median ± SD degree angle, respectively; *P* < 0.001) (Fig. 2*E*). To understand this further, we mapped their substitutions onto the HvEGT1 protein structure and compared them with the TM194 mutation. Interestingly, haplotype II causes an F391L substitution, just four amino acids away from TM194 (G395E) (*SI Appendix*, Fig. S7*C*) and both of these substitutions lie within a highly evolutionary conserved motif (position 391–400) shared by 37 plant species (*SI Appendix*, Fig. S8). In contrast, haplotype IV causes a S306C substitution, 89 amino acids upstream of the TM194 mutation (*SI Appendix*, Fig. S7*C*).

To investigate the effect of these mutations on EGT1 structure and function, we constructed a homology model for Tubby and F-box domains using Phyre2 (14) (*SI Appendix*, Fig. S7 *A* and *B*). For example, G395 sits in a highly positively charged cavity, likely to be stabilized by an adjacent negatively charged C-terminal site. The TM194 G395E substitution causes a small, neutral amino acid to be substituted by a larger, negatively charged residue, which is likely to destabilize this region and impact protein function. Furthermore, TM3580, a splice acceptor mutant containing a nine-amino acid deletion between residues 129 and 137, causes significant structural changes at the N-terminal region of the Tubby domain. This includes introduction of a short α-helical segment that presents amino acids with different physiochemical properties (polarity, hydrophobicity, and charge) on the domain surface (*SI Appendix*, Fig. S7 *D* and *H*). To understand the structure–function relation of these changes, we constructed the structure of the whole EGT1 protein using de novo prediction from the full protein sequence in AlphaFold2 (15) (*SI Appendix*). This structure shows that the F-box domain (*SI Appendix*, Fig. S7 *E* and *F*) presents a part charged, part hydrophobic protein–protein interaction interface to the Tubby domain (*SI Appendix*, Fig. S7 *E*–*H*). While the wild-type Tubby domain complements this physiochemical presentation (*SI Appendix*, Fig. S7*G*), the structural alterations in the TM3580 mutant leads to juxtaposition of negatively charged residues on the protein–protein interface (*SI Appendix*, Fig. S7*H*), likely destabilizing the overall structure and function of the mutant protein.

### *EGT1*-Mediated Root Growth Angle Regulation Is Conserved in Wheat.

Phylogenetic analysis of Tubby-like F-Box protein sequences in barley, wheat, rice, and brachypodium (*SI Appendix*) identified closely related proteins in all of these species (*SI Appendix*, Fig. S9). To address EGT1 function in another cereal, we screened (in silico) (*SI Appendix*) a TILLING population of tetraploid (AA BB) wheat cv. Kronos (16). Kronos2551 and Kronos3926 lines encoded premature termination codons in TRITD6Bv1G159700 (*HvEGT1* homeologous gene on wheat B genome) and the Kronos2708 line carrying a splice donor mutation in TRITD6Av1G172130 (*HvEGT1* homeologous gene on wheat A genome). Kronos2551 × Kronos2708 and Kronos3926 × Kronos2708 were crossed, then F1 plants were self-pollinated to create F2 plants. Progenies of selected wild-type and homozygous double mutants from two independent crosses were grown for 7 d in rhizoboxes for root growth angle analysis. Both the double mutants exhibited steeper seminal and lateral root growth angle compared with the progenies carrying wild-type alleles in both homeologs as well as homozygous mutations in just one homeolog (*SI Appendix*, Fig. S10). Hence, our results revealed that *TdEGT1* loci also control root growth angle in wheat and possibly other cereal and plant species.

### HvEGT1 Controls Root Growth Angle Via an AGO Mechanism.

**Fig. 3.**
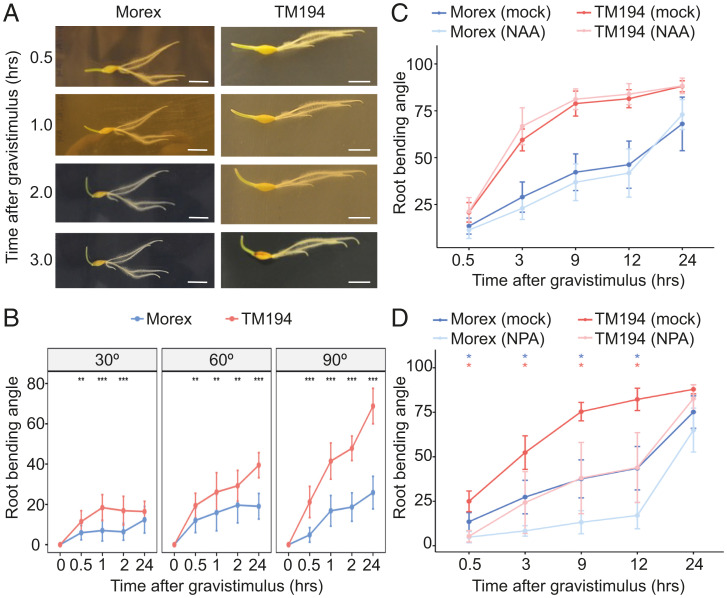
HvEGT1 controls root growth angle via auxin-independent AGO mechanism. (*A*) Representative images of root bending response of 4-d-old seminal roots in Morex and TM194 at 0.5, 1, 2, and 3 h after 90° tilting gravistimulus. (Scale bar, 1 cm.) (*B*) Measurement of dynamic change in root tip bending angle with increasing titling angle gravistimulations (from 30° to 60° to 90°) in Morex and TM194. (*C* and *D*) Auxin root bending sensitivity assay. Quantification of root bending response in Morex and TM194 at 0.5, 3, 9, 12, and 24 h after a 90° gravistimulus during (*C*) exogenous application of 10 nM NAA and (*D*) 1 µM auxin transport inhibitor NPA. An asterisk represents statistically significant difference, between treated and mock samples from respective genotype, assessed using Welch’s *t* test at *P < 0.05, **P < 0.01 and ***P < 0.001 in *n =* 2 independent replicates.

Different root classes adopt specific GSAs, which are maintained by competing gravitropic and AGO mechanisms (5, 6). Lugol staining of *Hvegt1* (TM194) mutant root tips revealed no observable differences in starch granule accumulation in statolith organelles, suggesting the root gravity-sensing machinery remains intact in the mutant (*SI Appendix*, Fig. S11). In *Hvegt1* mutants, seminal, lateral, and crown roots are no longer able to maintain their nonvertical GSA, suggesting *HvEGT1* operates as part of the AGO pathway. To validate this, we compared root bending responses of 4-d-old seminal roots in *Hvegt1* (TM194) and Morex after either a 30°, 60°, or 90° gravistimulus (Fig. 3*A*). If the gravitropic mechanism was compromised in *Hvegt1*, its root bending rate would be slower. In contrast, if the AGO mechanism was compromised in *Hvegt1*, the countering gravitropic mechanism would confer a higher bending rate. Our results revealed that *Hvegt1* roots exhibited a significantly higher bending angle and faster gravitropic response than Morex even after 0.5 h at a 30° tilting gravistimulus and this difference became even more exaggerated with increasing tilting angle (Fig. 3*B*). Hence, the *Hvegt1* mutant appears disrupted in its antigravitropic (rather than gravitropic) response, consistent with *HvEGT1* encoding a putative component of the AGO mechanism.

### HvEGT1 Appears to Function as Part of an Auxin-Independent AGO Mechanism.

Auxin transport and response have been reported to play a role in both gravitropic and AGO mechanisms (5, 8–10, 17), as exogenous application of auxin or auxin transport inhibitor influences GSA. We tested whether the *HvEGT1* expression and gravitropic bending response of *Hvegt1* mutant were influenced by exogenous auxin and auxin inhibitor treatment. qRT-PCR analysis (*SI Appendix*) revealed *HvEGT1* expression was not significantly induced after 10 nM 1-Naphthaleneacetic acid (NAA) and 1 µM 1-*N*-Napthylphthalamic acid (NPA) treatments in either Morex (*P* values 0.0818 and 0.0655, respectively) or TM194 mutant (*P* values 0.4043 and 0.2022, respectively) backgrounds (*SI Appendix*, Fig. S12).

**Fig. 4.**
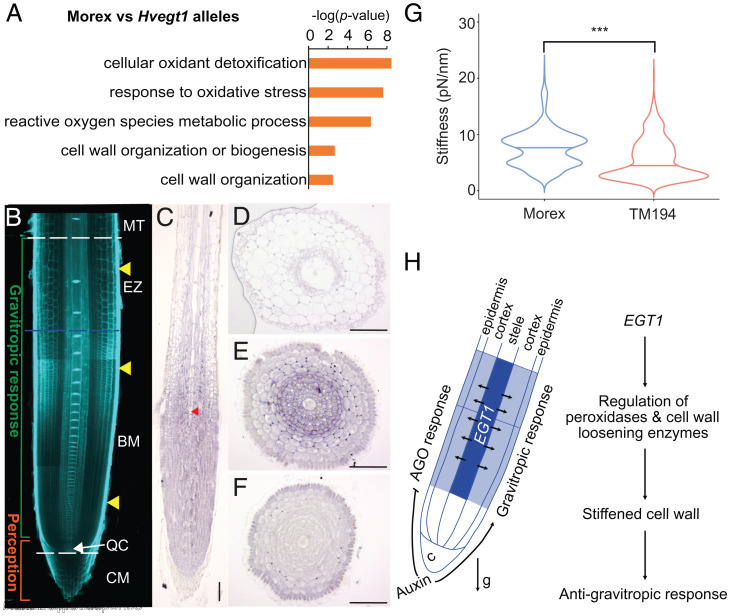
HvEGT1 transcriptionally regulates peroxidases and cell wall loosening enzymes and controls root cell wall stiffness. (*A*) Pruned version of GO enrichment of genes differentially expressed between Morex and both *hvegt1* mutant alleles (−1.5 < FC > 1.5; Benjamini–Hochberg FDR-corrected *P* < 0.05; FPKM ≥ 1). gProfiler (58) web server was used to perform GO enrichment analysis (settings: statistical domain space = all known genes; significance threshold = g:SCS, 0.05) (Dataset S3). GO enrichments were prefiltered using REVIGO (59) (revigo.irb.hr, default settings) to remove semantically redundant GO terms. Terms were pruned on REVIGO frequency (>0.25% and <2.5%) and the top five most significant GO categories visualized. (*B*) Schematic of gravitropic sensing and responding machinery in relation to root meristematic zones in barley cv. Morex: basal meristem (BM), columella (CM), elongation zone (EZ), maturation zone (MT), quiescent center (QC); blue line identifies the transition zone, yellow arrows highlight the approximate region where the cross-sections (*D*–*F*) were taken . (Scale bar, 100 µm.) (*C*) ISH on longitudinal section of root tips of cv. Morex with *HvEgt1* anti-sense probe. Red arrowhead shows exposed central metaxylem, suggesting root sections were in the center of the root. (*D*–*F*) ISH of root tip cross-sections in cv. Morex with *HvEGT1* antisense probe in EZ (*D*), higher BM (*E*), and lower BM (*F*). (Scale bar, 100 µm.) Additional replicates of Fig. 4 *C* and *E* are shown in *SI Appendix*, Fig. S17 *E* and *F*, respectively. (*G*) Force spectroscopy results showing stiffness values between Morex and TM194. Asterisks indicate ***P < 0.001 using nonparametric Wilcoxon test. (*H*) Schematic of the proposed model. Auxin-dependent gravitropic responses are known to function in outermost epidermal tissues, whereas auxin-independent AGO component *EGT1* functions in inner root cortical tissues temporally in basal meristem and transition zone. Dark and light blue color indicates the intensity of *EGT1* expression in these tissues and zones. We propose that EGT1 transcriptionally regulates peroxidases and cell wall loosening machinery and thus cell wall stiffness in root cortical tissues to counter gravitropic response to determine the GSA.

In contrast, auxin-inducible gene *HvIAA36* (18) showed a significant induction, both in Morex (*P* value 0.0091) and TM194 mutant (*P* value 0.0083) after 10 nM NAA treatment, suggesting that *HvEGT1* expression is auxin-independent. Additionally, the transcription factor binding site prediction tool PlantRegMap (19) did not identify any auxin response elements (AuxRE), which are required for auxin-dependent expression regulation within the 2.5-kb promoter of *HvEGT1* (Dataset S2). Similarly, root bending response at 0.5, 3, 9, 12, and 24 h after a 90° gravistimulus and NPA treatment significantly reduced root bending velocity to similar degrees in both mutant and wild-type, while no significant change was observed for NAA treatment (Fig. 3 *C* and *D*). This indicated that the auxin-mediated gravitropic response mechanism remains intact in *Hvegt1*. Consistently, our root RNA-seq dataset did not show overrepresentation of auxin signaling genes in either *Hvegt1* mutant alleles compared to wild-type (Fig. 4*A* and Datasets S3 and S4). Furthermore, detailed comparative expression analysis of auxin transport and biosynthesis genes showed that auxin signaling pathway in both mutant alleles remain mostly unperturbed when compared to Morex (Dataset S5). Taken together, our root bioassays, promoter analysis and RNA-seq results suggest that *HvEGT1* functions as part of an auxin-independent AGO mechanism.

### Mutations in *HvEGT1* Deregulates Expression of Reactive Oxygen Species Homeostasis and Cell Wall Enzymes.

To determine why *Hvegt1* roots bend more rapidly than wild-type, we analyzed root transcript profiles to reveal which classes of genes were differentially expressed. In total, 6,443 genes were identified to be differentially expressed (Benjamini–Hochberg false-discovery rate [FDR] corrected *P* < 0.05, −1.5 < fold-change [FC] > 1.5 and fragments per kilobase of transcript per million mapped reads [FPKM] > 1) between comparisons of TM194 vs. Morex, TM3580 vs. Morex, and TM194 vs. TM3580 (Dataset S3). We focused on the 841 differentially expressed genes in both *Hvegt1* mutant alleles compared to Morex. Gene ontology (GO) enrichment identified overrepresentation for mainly hydrogen-peroxide and cell wall-related biological processes (Fig. 4*A* and Dataset S4). Interestingly, hydrogen peroxide catabolic and metabolic processes were explicitly enriched by 21 cell wall peroxidases (Dataset S6). This suggested that *Hvegt1* mutant alleles may have differences in ROS homeostasis compared to Morex. Consistently, reactive oxygen species (ROS) detection assays using CM-H_2_DCFDA revealed that the *Hvegt1* (TM194) mutant (*SI Appendix*), when compared to Morex, has a reduced level of ROS in root tips and explicitly in the root meristem and elongation zone (*SI Appendix*, Fig. S13). Peroxidases are associated with cell wall loosening and stiffening processes through ROS for oxidative polymerization of cell wall aromatic compounds within phenolics or oxidative scission of cell wall polysaccharides (cellulose, hemicellulose, e.g., xyloglucans and pectins) (20). Consistently, we observed that cell wall organization or biogenesis, including xyloglucan metabolic processes, were enriched by 23 genes encoding cell wall-modifying enzymes (i.e., expansins, chitinase family proteins, glucosyltransferases, pectin methylesterase inhibitors, fasciclin-like arabinogalactan proteins, and xyloglucan hydrolases) (Dataset S6). Many of these enzymes modify cell wall components during growth and development (21, 22). Coexpression analysis with published barley RNA-seq data (23) further indicated that several cell wall gene modules were differentially expressed in *hvegt1* versus wild-type roots (*SI Appendix*, Fig. S14). The spatiotemporal expression enrichment of orthologs of these peroxidases and cell wall in rice roots (24, 25) revealed that the majority are mostly expressed in stele tissues of proximal meristem and elongation zones (*SI Appendix*, Fig. S15).

### *HvEGT1* Is Highly Expressed in Expanding Root Tissues.

To determine the site of action of *HvEGT1*, we elucidated its spatial expression in root tissues using RNA in situ hybridization (ISH) (26). An *HvEGT1* specific, nonconserved region (compared to other barley Tubby genes) spanning the end of the CDS and the start of the 3′ untranslated region (UTR) was used to design and synthesize digoxigenin-labeled antisense and sense probes (*SI Appendix*, Fig. S16). ISH longitudinal and radial root sections revealed the *HvEGT1* transcript is most highly abundant in basal meristem and transition zone cells (Fig. 4 *B* and *C* and *SI Appendix*, Fig. S17). No major difference was detected for *HvEGT1* transcript in ISH roots of TM194 mutant compared to Morex (*SI Appendix*, Fig. S18), consistent with our RNA-seq and qRT-PCR results (*SI Appendix*, Fig. S6). The level of *HvEGT1* expression then decreased until it became undetectable in maturation zone cells. The hybridized cross-sections revealed highest *HvEGT1* transcript levels in stele and cortical tissues in the basal meristem and elongation zones. In contrast, sections through the apical meristem showed only a weak signal (Fig. 4 *D*–*F*). Hence, *HvEGT1* expression is primarily associated with root cells starting to elongate, consistent with the spatiotemporal expression of the classes of genes identified to be differentially regulated in our *Hvegt1 vs.* wild-type RNA-seq analysis (*SI Appendix*, Fig. S15). The enriched pattern of EGT1 expression (and differentially expressed ROS and cell wall genes) in stele and cortical root elongation zone tissues is distinct from the outermost tissues known to be involved in root gravitropic bending response (27).

### Atomic Force Spectroscopy Suggests *Hvegt1* Mutants Have Less Stiff Root Cell Walls.

Given that loss of *HvEGT1* deregulates genes encoding cell wall-modifying enzymes, we examined whether EGT1 regulates cell wall properties and, hence, cell wall stiffness. To test this hypothesis, we analyzed 50-µM-thick longitudinal cross-sections of 4-d-old seminal root tips of Morex and TM194 mutant using force spectroscopy under plasmolysed but hydrated conditions (28, 29). Specifically, we characterized nine independent areas within the elongation zone in a 3 × 3 array (*SI Appendix*, Fig. S19*A*), performing 100 < *n* < 360 indentation curves for each biological replicate (Morex = 4 and TM194 = 5). The obtained force versus distance curves were used to determine apparent stiffness (pN/nm) (*SI Appendix*, Fig. S19*B*). Morex roots exhibited an average stiffness of 7.60 ± 3.30 pN/nm, while TM194 showed 5.6 ± 3.60 pN/nm (Fig. 4*G*). Our results suggest that there is a significant reduction (26.32%, *P* < 0.001) in cell wall stiffness in elongating cells of the *Hvegt1* mutant compared to wild-type. Interestingly, when analyzed 3 × 3 array datasets were subdivided into stele versus cortical tissues, mutant roots have a significantly lower stiffness in root cortical tissues (35.75%, *P* < 0.001), while there was no significant difference for stele tissues (*SI Appendix*, Fig. S19*C*). Hence, reduced cell wall stiffness (notably in the cortical layers) in *hvegt1* mutant roots is likely to disrupt their ability to counteract gravitropic bending, causing them to grow steeper along a gravity vector.

## Discussion

Root angle is a key trait in crops to ensure efficient capture of soil resources such as water and nutrients. Although recent studies have identified major quantitative trait loci associated with seminal root angle by genome-wide association studies based on phenotyping of different barley genomic populations (30, 31), knowledge about the underlying genes controlling root angle in barley remains limited. A limited number of root angle regulatory genes have been identified in other cereals, including *DRO1* (1), *VLN2* (32), *PIN2* (33), *RMD* (3), and *CIPK15* (2). To address this knowledge gap, we characterized a chemically mutagenized population of the cv. Morex (11) for a steeper seminal root phenotype, where we identified the TM194 mutant that exhibited steeper growth angle not only for seminal roots but also for lateral and crown roots. Genetic and genomic approaches revealed that a mutation in the *EGT1* gene is responsible for the steeper root angle phenotype.

*HvEGT1* encodes a Tubby-like protein (TLP) that contains conserved C-terminal Tubby and N-terminal F-box domains (34, 35). Tubby domain-containing proteins are proposed to act as bipartite transcription regulator (36, 37), whereas F-box proteins facilitate protein ubiquitination by acting as bridges between specific substrates and the components of the SCF-type (Skp1-Cullin-F-box) or ECS-type (ElonginC-Cullin-SOCS-box) E3 ubiquitin ligase complexes (34, 38). Previous mutant studies in *A. thaliana* have identified that TLPs AtTLP3 and AtTLP2 could play roles in regulation of ROS signaling and cell wall-related genes, respectively (39, 40). Consistent with this, our transcriptome analysis identified that ROS homeostasis and cell wall-modifying enzymes are deregulated in mutants compared to wild-type, suggesting that some of these genes may represent downstream targets of *HvEGT1*. Protein–protein interaction database analysis suggests EGT1 might regulate proteins involved in cell elongation and cell expansion by regulating cell wall-modifying enzymes or cell wall material synthesis or transport (*SI Appendix*, Fig. S20). Further work will be required to pinpoint whether these are direct or indirect regulatory targets of EGT1. Kirschner et al. (41) recently reported a barley mutant with a steeper root growth angle phenotype termed *ENHANCED GRAVITROPISM 2* (EGT2), whose wild-type gene encoded a STERILE ALPHA MOTIVE-containing protein and also deregulates cell wall-related genes. Although *EGT1* and *EGT2* both function in auxin independent AGO mechanisms, they are expressed in distinct tissue types and target different set of cell wall genes. Additionally, no change in *EGT1* expression was observed in *Hvegt2* mutant and vice versa (*SI Appendix*, Fig. S21). Hence, *EGT1* and *EGT2* could function in parallel AGO pathways to control root angle in barley and wheat.

How does *EGT1* control root angle? *EGT1* expression is detected in stele and cortical cells in the root meristem and elongation zones (Fig. 4*C*), which overlaps with cortical cell wall stiffness differences detected using AFM in wild-type versus *Hvegt1* mutant root tips (*SI Appendix*, Fig. S19). Interestingly, *Hvegt1* mutant root tips also show a reduction in ROS levels where *EGT1* is normally expressed (*SI Appendix*, Fig. S13). ROS triggers cell wall cross-linking and increases stiffness (42). It is plausible that EGT1 functions to regulate ROS homeostasis in cortical tissues to control optimal stiffness required for maintaining roots at specific GSAs. EGT1-dependent stiffening of cortical cell walls may serve to counteract the gravitropic machinery’s known ability to bend roots via the outermost epidermal tissues (27). However, in the absence of EGT1, cell walls of root cortical tissues are less rigid, enabling the gravitropic machinery to bend the *Hvegt1* mutant root much more rapidly. Hence, we propose that auxin-dependent gravitropic bending operates in outer epidermal tissues, while auxin-independent EGT1-mediated stiffening mechanisms operate in root cortical tissues. Such a dual auxin-dependent/independent mechanical model for regulating root gravitropic bending rate also provides a simple mechanism for explaining GSA, where the relative strength of the auxin-dependent gravitropic and EGT1-dependent antigravitropic pathways operating in outer tissues (epidermis and cortex, respectively) could determine set-point angle in different root classes.

Could new crop varieties with altered root angle be selected using *EGT1*? Loss of function *EGT1* alleles exhibit very steep angles for all root classes, likely causing them to inefficiently compete with each other for resource capture. However, results from haplotype analysis appear more promising since nucleotide polymorphisms within the *HvEGT1* sequence were observed to determine natural variation in root growth angle in a barley diversity panel. Hence, selecting or engineering *HvEGT1* alleles to adapt cultivars for specific environmental conditions, such as different soil types or variable water table depth, would appear possible. Further studies targeting EGT1 promise to open novel avenues for developing bespoke crop varieties with optimized root system architecture for efficient resource capture.

## Materials and Methods

### Plant Material.

Barley *Hvegt1* mutant alleles (TM194 and TM3580) from the TILLMore barley mutant population (11), wheat *Tdegt1* mutant alleles (*Tdegt1_wtA/mutB*, *Tdegt1_mutA/wtB*, and *Tdegt1_mutA/mutB*) from a wheat TILLING population described in Krasileva et al. (16), and respective wild-types (cv. Morex and cv. Kronos) were used for root growth angle imaging and measurement analyses on flat screens using semihydroponic systems and in soil using rhizotrons and X-ray microCT and X-ray CT. An F2 population obtained by crossing TM194 mutant and another barley wild-type cv. Barke was used for BSA. TM194 and TM3580 mutant alleles were used for the WGS experiment and mapped to Morex v.1 reference genome. TM194 mutant allele was used for exome sequencing experiment. Morex and *HvEGT1* mutant alleles (TM194 and TM3580) were used for RNA-seq analysis and their wild-type and mutated protein sequences, respectively, were used for protein structure analysis (*SI Appendix*). Morex and TM194 mutant were used for shoot and leaf growth angle quantification (*SI Appendix*), gravistimulus-induced root bending assays (on mock, NAA, and NPA supplemented media), Lugol’s iodide staining (*SI Appendix*), qRT-PCR analysis (*SI Appendix*), H_2_DCFDA ROS detection assay (*SI Appendix*), and AFM spectroscopy experiments. Selected lines from the barley WHEALBI diversity panel (https://www.whealbi.eu) were used for haplotype network analysis and root growth angle measurements.

### Barley and Wheat Two-Dimensional Root Phenotyping.

#### Semihydroponic system.

For the semihydroponic system, seeds were washed in 70% ethanol for 1 min, then in 1% sodium hypochlorite + 0.02% Triton X-100 for 5 min and rinsed with distilled water. Sterilized seeds were pregerminated for 24 h at 28 °C in wet filter paper. Equally germinated seeds were placed between two sheets of 50 × 25 cm of filter paper (Carta filtro Labor, Gruppo Cordenons SpA) soaked in demineralized water, rolled, positioned vertically in a 5-L plastic beaker with 1 L of demineralized water. Barley seedlings were grown for 10 d at 24 °C and wheat seedlings were grown for 7 d at 22 °C with a 16/8-h photoperiod. Root growth from both experiments were imaged using a DSLR camera and vertical root angle for seminal and lateral (from the insertion with the seminal root) roots were calculated using ImageJ software.

#### Two-dimensional soil experiments.

For two-dimensional soil experiment purposes, barley and wheat *egt1* mutants and their respective wild-types were grown up to 20 d in the GrowScreen-Rhizo rhizotrons automated platform and analyzed as previously described (43).

### Barley Nondestructive 3D Root Phenotyping.

Nondestructive 3D phenotyping was performed on Morex and TM194 using X-ray microCT and X-ray CT (*n* = 6 independent replicates).

#### X-ray microCT.

For X-ray microCT, seeds were pregerminated in Petri dishes for 1 d at 21 °C in dark. Successful seedlings with equally germinated roots were grown in PVC columns (8-cm diameter × 15-cm height) filled with sandy loam soil from the University of Nottingham experimental farm field, sieved at <2 mm and maintained at notional field capacity moisture until 9 DAG. Each column was scanned using a Phoenix v|tome|x M 240-kV X-ray microCT scanner (Waygate Technologies [a Baker Hughes business]) at the Hounsfield Facility (University of Nottingham, Sutton Bonington Campus, United Kingdom). The voltage and current were set at 180 kV and 180 µA, respectively. A voxel resolution of 55 μm was used in all scans. During the scan, the specimen stage rotated through 360° at a rotation step increment of 0.166° collecting a total of 2,160 projection images. Each image was the integration of four frames with a detector exposure time of 250 ms, resulting in a 75-min scan time. A 0.1-mm copper filter was applied to the front of the exit window of the X-ray tube during the scan to reduce beam hardening artifacts.

#### X-ray CT.

For the X-ray CT, well-watered plants were grown in larger PVC soil columns (20-cm diameter, 100-cm height) until full maturation stage. Each column was then scanned using a Phoenix v|tome|x L Custom 320-kV X-ray CT system (Waygate Technologies) at the Hounsfield Facility (University of Nottingham, Sutton Bonington Campus, United Kingdom). The voltage and current were set at 290 kV and 6,200 µA, respectively. A voxel resolution of 150 μm was used in all scans. During the scan, the specimen stage rotated through 360° at a rotation step increment of 0.15° collecting a total of 2,400 projection images. To reduce image noise, each projection image was an integration of 12 frames with a detector exposure time of 131 ms. Each scan took ∼240 min. A 1-mm copper filter was applied to the exit window of the X-ray tube and a further 0.5-mm Cu filter applied over the detector panel to reduce beam hardening artifacts.

For all CT images, the scans were reconstructed using DatosRec software (Waygate Technologies, Baker Hughes Digital Solutions). Radiographs were visually assessed for sample movement before being reconstructed in 16-bit depth volumes with a beam hardening correction of 8. An inline median filter was applied to reduce noise in the image of the CT X-ray data. Reconstructed volumes were then postprocessed in VGStudioMAX (v2.2.0; Volume Graphics). Root system architecture was first segmented from the reconstructed volumes using the polyline tool within VGStudioMAX and then quantified using an in-house software tool called PAM (Polyline Angle Measurement). PAM extracts the 3D coordinate points (2 to 5 *XY* slices apart) for each polyline and translates these into a 3D model. The angle of each polyline (root) is calculated from the difference of a vertical vector from the position of the uppermost coordinate point of the polyline (e.g., the soil surface). Therefore, steeply growing roots have a low angle value and shallow roots have a large angle value. Measurement of root angle was terminated once the root has touched or interacted with the pot wall to avoid any physical interference on undisturbed root angle.

### Bulked Segregant Analysis.

BSA was carried out on F2 plants derived from the cross TM194 × cv. Barke which were grown in flat rhizotrons, each composed by a rigid 38.5 × 42.5-cm black plastic screen and by two wet filter paper sheets. Seeds were disinfected for 5 min in a 1.2% solution of sodium hypochlorite and incubated for 24 h at 28 °C. Five pregerminated seeds per rhizotron were placed between two filter paper sheets. Rhizotrons were vertically positioned inside a plastic tank filled with deionized water to reach a level of 5 cm from the bottom and put in a growth chamber with a 16/8-h photoperiod and a temperature of 22°/18 °C for 13 d. After that period, root growth angles were measured, and seedlings were divided into wild-type and mutant phenotype groups. Fifteen plants from each group were selected for single plant DNA extraction. Leaves were lyophilized and foliar samples of ∼2 cm^2^ were homogenized for 3 min in a TissueLyser. DNA was extracted with the Macherey-Nagel Nucleospin Plant II kit and quantified with NanoDrop. Two DNA bulks, steeper and wild-type root angle phenotype were prepared in double, mixing equal amounts of each plant and bringing to a final concentration of 50 ng/μL, in addition to single plant DNA from 10 plants showing steeper angle and all sample were genotyped with the 9k Illumina Infinium iSelect barley SNP array. The results were analyzed with GenomeStudio (Illumina), and Δ/θ values used as index of allele proportion at each SNP marker. Δ/θ Values were calculated as the squared difference between the theta value of wild-type and steeper angle phenotype bulk.

### Whole Genome and Exome Sequencing.

Genomic DNA for WGS of the two mutants TM194 and TM3580 was prepared as described above and sequenced with Illumina HiSeq PE150, obtaining 727,190,417 paired-end reads for an average coverage of ∼23× for TM194 and 792,713,857 paired-end reads for an average coverage of ∼25× for TM3580. Reads were aligned to Morex v1 reference sequence (44) with BWA v7.12 (45) and variants in the genomic space were called with SAMtools v1.3 (46), filtering for a minimum read depth of 5×, PHRED quality > 40. To discard background mutations due to the differences between the Morex reference sequence and the Morex parental seeds that had previously been used in the mutagenesis, the SNP calling for TM194 considered an additional eight TILLMore mutant WGS data that were available at that moment, filtering with a custom AWK script for a minimum ratio DV/DP of 0.8 for the *Hvegt1* mutants and a maximum ratio of 0.2 in every other mutant, where DP is the coverage depth at the SNP position and DV is number of nonreference bases at the same position. SNP effects were predicted with SNPEff v3.0.7 (47). TM194 mutant was predicted to harbor a missense substitution within fourth exon while TM3580 mutation at the end of first intron was predicted to cause splice-acceptor variant (Dataset S1). TM194 exome sequencing was carried out as described in (13).

### Haplotype Analysis of *HvEGT1* in WHEALBI Barley Germplasm Collection.

A haplotype analysis of SNP data from the barley diversity panel WHEALBI (48), consisting of 459 barley accessions, of which 199 are cultivars, 202 landraces, and 4 wild, was conducted in the coding region of *HvEGT1*. Files were imported into R Studio and package *pegas* (49) v0.14 was used to detect haplotypes. Six haplotypes were found. The MUSCLE multialignment was produced with Mega X v10.2.4 (50) and exported to the NEXUS format (51). The haplotype TCS network (52) was produced with PopART (popart.otago.ac.nz).

### Gravity and Auxin Sensitivity Bioassays.

Seedlings of Morex and TM194 mutant were pregerminated for 2 d in dark at 21 °C. Equally germinated seeds were then transferred on 12-cm^2^ plates containing 1% agar media and grown 1 to 2 d at 21 °C with 12/12-h photoperiod. For the gravity response bending bioassay, plates were then rotated by 30°, 60°, and 90° and then images were collected at multiple timepoints using a Nikon D5100 camera. For the auxin sensitivity assay, seedlings were then transferred to mock, 10 nM NAA and 1 µM NPA media for 2 h before rotating plates by 90°. The time-lapse image stack was then generated by taking images every 30 min for 12 h in dark and then once at 24 h after gravistimulus using the robotic imaging facility at the University of Nottingham. Root tip bending angle was the quantified using FIJI (53).

### RNA-Seq and Data Analysis.

Seeds of Morex, TM194, TM3580 genotypes were sterilized with 0.5% sodium hypochlorite solution for 5 min followed by five washes of sterile water. Sterilized seeds were germinated on sterile Whatman filter paper placed in a Petri dish for 2 d at 21 °C in dark. Equally germinated seeds were vertically grown on 1% agar plates for 2 d at 22 °C, 16/8-h photoperiod. Root tips from seminal roots growing on media surface were dissected at the first visible root hairs and samples were snap frozen using liquid nitrogen and then stored at −80 °C. Root tips from 15 seedlings were pooled together per replicate and RNA extraction was then performed using TRIzol and Rneasy mini kit (Qiagen) for RNA-seq analysis. For each genotype, four biological replicates were prepared.

Library preparation and Illumina sequencing was performed by Novogene Company Limited. RNA-seq was performed on an Illumina HisEq. 2000 platform and 150-bp paired-end reads were generated according to Illumina’s protocol. Data analysis was performed by standard Novogene bioinformatics pipeline. Raw reads were first processed to remove adapter and poly-N sequences and low-quality reads. High quality paired-end clean reads were mapped to reference genome IBSC_v2 using HISAT2 (54) software. Cufflinks Reference Annotation Based Transcript (RABT) assembly method (55) was used to assemble the set of transcript isoforms of each bam file obtained din the mapping step. HTSeq (56) was used to count the read numbers mapped of each gene, including known and novel genes. FPKM of each gene was calculated based on the length of the gene and reads counts mapped to this gene. The hierarchical cluster analysis of gene expression among replicates indicated poor correlation for one of the four replicates for sample TM194, which was removed from further analysis. Differential expression analysis between TM194 vs. Morex, TM3580 vs. Morex and TM3580 vs. TM194 was performed using DESeq2 (57) R package. The resulting *P* values were FDR-corrected using the Benjamini and Hochberg’s approach and genes with an adjusted *P* < 0.05, −1.5 < FC > 1.5, and FPKM > 1 were assigned as differentially expressed. GO enrichment was performed using gProfiler (58) web server with settings (statistical domain space = all known genes; significance threshold = g:SCS, 0.05). REVIGO (59) (revigo.irb.hr, default settings) was used to remove semantically redundant GO terms.

### Barley RNA ISH.

RNA ISH was performed to target *HvEGT1* expression in Morex. Seeds of barley cultivar Morex were surface sterilized in 20% (vol/vol) sodium hypochlorite solution for 10 min, then rinsed with MilliQ water five times before pregermination overnight. Seeds were then placed into germination pouches (Phytotc) for 5 d. Fresh root tips (2 cm) were harvested and fixed in formalin-acetic acid-alcohol (FAA) (50% [vol/vol] 100% ethanol, 5% [vol/vol] glacial acetic acid, 25% [vol/vol] 16% paraformaldehyde (electron microscopy grade), 20% [vol/vol] diethyl pyrocarbonate [DEPC]-H_2_O, 0.1% [vol/vol] Tween 20). Root tips with FAA were placed on ice for 2 h including 15 min of vacuum infiltration, followed by two 10 min washes in 70% ethanol/DEPC-H_2_O, and then stored at 4 °C overnight. The samples were dehydrated and cleared with a series of ethanol and Histochoice washes before being embedded in molten paraffin wax. The embedded samples were stored at 4 °C under Rnase free conditions before sectioning. The paraffin wax blocks with the root samples were sectioned at 7-μm thickness using a Leica microtome and mounted onto poly-l-lysine–coated slides prior to ISH. Digoxigenin-labeled antisense and sense probes were designed and synthesized as shown in *SI Appendix*, Fig. S10. The probes specific to *HvEGT1* were amplified from Morex root cDNA, using primers fused with the T7 promoter sequence at the 5′ end to allow in vitro transcription. The probes were designed to recognize the end of the coding sequence and 3′UTR of the gene. The barley histone H4 gene was used as a positive control. The ISH and detection were performed using the InsituPro Vsi robot (Intavis) (26).

### Force Spectroscopy Using AFM.

#### Sample preparation.

Root tips from 4-d-old seedlings of Morex and TM194 were grown in 1% agar (Scientific Laboratory Supplies) at 23 °C, 16/8-h daylight/darkness. Root tips from seminal roots were harvested at 1.5 cm, set in 5% agarose (Sigma-Aldrich) creating 2-cm × 1-cm blocks for cross-sectioning. Longitudinal cross-sections of 50 µm were obtained using a vibratome (frequency 50 Hz, amplitude 1 mm) (7000smz-2, Campden Instruments) and observed using light microscopy to confirm stele and cortical tissues were correctly exposed with visible elongation zone. Specimens were then stored in de-ionized water at 4 °C overnight and analyzed by AFM 1 d after preparation.

#### AFM mechanical analysis.

A Dimension ICON (Bruker Nano) using dedicated software (Nanoscope 9.4) was used probe all root samples. MLCT-E (Bruker Nano) cantilevers were used across all analyzed samples. Before mounting the MLCT-E cantilever, all other cantilevers on the same AFM probe were removed using fine tweezers guided by a binocular. This was performed to avoid parallel probes causing localized sample surface movement interfering with the indentation measurements. AFM probes were then mounted and secured to a fluid cell (DECAFMCH-PFT, Bruker Nano) and calibrated in de-ionized water before analysis. The average spring constant of cantilevers used in experiments was 0.008 ± 0.002 N/m. ​Root sections in agarose were fixed to glass slides using UHU Plus 2-min curing glue (Bolton Adhesives) on the exterior of agarose only and hydrated using de-ionized water for 30 min before AFM analysis. Operating in force-spectroscopy mode under water-hydrated conditions, nine independent areas were monitored within the observable elongation zone in a 3 × 3 array shown in *SI Appendix*, Fig. S19*A*. Indentations were performed in the observable center of root meristem cells on each section generating a total of 100 < *n* < 360 force curves for each biological replicate (Morex = 4, TM194 = 5). Using dedicated software (Nanoscope Analysis 1.9), apparent stiffness (pN/nm) values were obtained from individual force-distance curves using a contact point based fit and linear stiffness model. Data from each area was pooled and analyzed using a nonparametric Wilcoxon test for significant differences between sample type and areas (*P* < 0.001). Additionally, data from these nine areas were categorized into cortical and stele tissues and the results of comparison between Morex and TM194 are shown in *SI Appendix*, Fig. S19*C*.

## Supplementary Material

Supplementary File

Supplementary File

Supplementary File

Supplementary File

Supplementary File

Supplementary File

Supplementary File

## Data Availability

All study data are included in the main text and supporting information.
