# Supplementary material for: Root angle is controlled by EGT1 in cereal crops employing an antigravitropic mechanism
Source: Proc Natl Acad Sci U S A. 2022 Jul 26;119(31):e2201350119. doi: 10.1073/pnas.2201350119 (PMC9351459; doi:10.1073/pnas.2201350119)
Supplement: Supplementary File [file pnas.2201350119.sapp.pdf]

PNAS

Supplementary Information for

Root angle is controlled by EGT1 in cereal crops employing a novel anti-gravitropic mechanism.

Riccardo Fusi[1,2,#], Serena Rosignoli[3,#], Haoyu Lou[1,4,5,#], Giuseppe Sangiorgi[3], Riccardo Bovina[3], Jacob K. Pattem[6], Aditi N. Borkar[7,8], Marco Lombardi[1,9], Cristian Forestan[3], Sara G. Milner[3], Jayne L. Davis[1], Aneesh Lale[1,2], Gwendolyn K. Kirschner[10], Ranjan Swarup[1], Alberto Tassinari[3], Bipin K. Pandey[1,2], Larry M. York[1,11], Brian S. Atkinson[1], Craig J. Sturrock[1], Sacha J. Mooney[1], Frank Hochholdinger[10], Matthew R. Tucker[4,5], Axel Himmelbach[12], Nils Stein[12,13], Martin Mascher[12,14], Kerstin A. Nagel[15], Laura De Gara[9], James Simmonds[16], Cristobal Uauy[16], Roberto Tuberosa[3], Jonathan P. Lynch[1,17], Gleb E. Yakubov[6], Malcolm J. Bennett[1,2,*], Rahul Bhosale[1,2,*] & Silvio Salvi[3,*]

***Corresponding authors**: Malcolm Bennett, Rahul Bhosale & Silvio Salvi

Email: malcolm.bennett@nottingham.ac.uk, rahul.bhosale@nottingham.ac.uk and silvio.salvi@unibo.it

This PDF file includes:

Supplementary text
Figures S1 to S21
Table S1
Legends for Datasets S1 to S6
SI References

Supplementary Information Text

Methods

Shoot and leaf growth angle measurements

For shoot growth angle measurements, plants of TM194 and Morex were grown in blue papers for 7 DAG with a day temperature of 21°C (16 h) and a night temperature of 18°C (8 h). Leaf growth angles were measured using the angle tool in FIJI. *N*=8 plants per genotype were used. *For leaf growth angle measurements*, plants of TM194 and Morex were grown in the greenhouse, in a peat and vermiculite growing medium (Vigorplant Irish and Baltic peat-based professional mix) in 15 × 15 × 30 cm polyethylene pots with a day temperature of 22°C (16 h) and a night temperature of 18°C (8 h). Greenhouse lighting was a mix of natural light supplemented with artificial light by 400-watt high-pressure sodium lamps (Sylvania SHP-TS 400W Grolux). Leaf growth angles were measured for the first three leaves of each plant, including the flag leaf, at flowering time (Zadoks growth stage 6). A goniometer was used to measure the angle between the proximal region of the adaxial surface of the blade and the stem.

HVEGT1 structure modelling and *Hvegt1* mutant allele mapping

The protein sequence obtained by translating Transcript 3 (427 aa) from *HvEGT1* (*HORVU6Hr1G068970*) entry was used to construct a homology model using the Phyre2 (1) server. A homology modelling approach was chosen over *de novo* structure prediction from first principles as the gene of interest was inferred to have F-box and Tubby-Like domains, which were confirmed by the protein domain analysis using EBI Interproscan tool. Tubby-Like domain was alone used in the structure prediction algorithm. WGS and haplotype analysis identified mis-sense amino acid substitutions (TM194 and Haplotype II and IV, respectively) were mapped on the predicted structure. Splice acceptor mutation (TM3580) was also visualised with respect to organised F-box and Tubby-like domains. Protein sequence was further studied for its conservation to function prediction across plant species using ConSurf algorithm.

Wheat EGT1 mutant identification

Durum wheat (*Triticum turgidum*) *Tdegt1* mutants were identified from a TILLING population developed in tetraploid *cv* Kronos (2). Two selected lines (Kronos2551 and Kronos3926) carrying premature termination codons in TRITD6Bv1G159700, the *TdEGT1* homoeologous gene on the B genome (*TdEGT1_wtA/mutB*), were both crossed with the line Kronos2708, carrying a splice donor mutation in TRITD6Av1G172130, the *TdEGT1* homoeologous gene on the A genome (*TdEGT1_mutA/wtB*). F1 plants obtained from both crosses were self-pollinated. Progenies of selected wild-type, single and double mutant F2 individuals derived from the two independent initial crosses (*TdEGT1_mutA/mutB*) were grown in semi-hydroponic system and analysed for seminal root angle analysis as mentioned above.

Phylogenetic analysis of Tubby-like F-Box Protein Sequences in selected monocots

HvEGT1 was used as a seed gene to select orthologous genes (>40% identity) from key monocot species such as barley (*Hordeum Vulgare*), wheat (*Triticum turgidum*), rice (*Oryza Sativa spp. Japonica*), maize (Zea Mays B73) and brachypodium (*Brachypodium distachyon*) using interactive phylogenetic module of Monocots Plaza 4.5 (3). Protein sequences were aligned using MUSCLE and tree was constructed using FastTree algorithm. Generated Newick file was imported into iTOL to create an unrooted tree.

Lugol's staining assay

To visualise statoliths in root tips of Morex and TM194 mutant, 1 day pregerminated seedlings were grown in paper rolls in 21 ºC, 16/8 daylight photoperiod growth conditions for 5 days. 1 cm root tips were then embedded in 10% low melting point agarose and sliced using vibratome (7000 smz-2, Campden Instruments, UK) set as 50 Hz frequency, 1 mm amplitude and 40 µm section. Sections were stained using Lugol's iodine solution (VWR chemicals) for 3 minutes and then visualised using LEICA DM 550B light microscope.

RTqPCR analysis during NAA and NPA treatments

Morex and egt1 mutant TM194 seeds were sterilized with 70% ethanol for 5 mins then 15% bleach for 5 min and washed 3-5 times with distilled water. Sterilised seeds were sown directly on ½ Hoagland's No. 2 Basal salt (Sigma, H2395), 1% agar plates and plates were kept at 4ºC for 5 days to improve germination rate. Plates were then transferred to growth room with 16/8h photoperiod and temperature of 22°/18°C. 3-day old plants (post germination) were then transferred to plates containing ½ Hoagland's solution, 1% agar, 0.1% DMSO, plus either 10nM NAA or 1μM NPA. Root tips (5mm form tip) from > 3 individual plants (i.e., ~15 plants) were pooled at 0h and 8h post transfer and flash frozen in liquid nitrogen. RNA was extracted using the Monarch® Total RNA Miniprep Kit (NEB, T2010S) as per protocol and cDNA prepared using Thermo Scientific Revertair frist strand cDNA synthesis kit. Quantitative RT-PCR (qRT-PCR) analysis was carried out with SYBRgreen (Meridian bioscience, Sensimix SYBR Hi-ROX Kit) using qTower 384G machine (Analytikjena). *HvAlpha-Tub* (*HORVU1Hr1G081280.1*) and *HvGADPH* (*HORVU6Hr1G054520*) were used as internal control and auxin responsive genes *HvIAA36* (HORVU0Hr1G021630.1), *HvIAA22* and *HvIAA20* primers used from Shi et al. (4) for primers see Supplementary Table 1. Three independent biological repeats with four technical replicates were used. Data was analysed using delta Ct method and statistical analysis carried out using Student's *T*-test. Each treated sample per genotype was normalised by respective DMSO sample.

ROS detection assay

TM194 mutant and Morex seeds were surface sterilised using 20% (v/v) bleach for 4 minutes and were then rinsed five times with de-ionised water. Washed seeds were then germinated on a filter paper saturated with de-ionised water in a petri dish kept at 21 ºC for 48 hours. Seedlings with uniform growth were placed on a germination paper, rolled into paper rolls and grown vertically at 21 ºC for 4 days. CM-H_2DCFDA (Sigma-Aldrich) dissolved into dimethyl sulfoxide (DMSO, VWR Life Science) was used to visualize the localization of ROS in Morex and TM194 mutant root tips. 20 μM CM-H_2DCFDA was prepared in 50 μM potassium chloride buffer (50mM KCl, 10mM MES, pH 6.0) on the day of the experiment. Root samples were taken 1 cm from the tip and were treated with 1 ml of CM-H2DCFDA for 15 minutes under vacuum. After treatment, samples were washed thoroughly with potassium chloride buffer four times. Samples were then placed on a glass slide with 50% glycerol as mounting agent and visualized with the Zeiss Leica DM5000 fluorescent microscope. CM-H_2DCFDA could be deacetylated by cellular esterase and then subject to oxidisation by ROS to 2',7'-dichlorofluorescein (DCF), which is highly fluorescent and could be detected under excitation and emission spectra of 492-495 nm and 517-527 mm, respectively. To minimize any variation in processing and imaging samples, all roots per seedling were stained, mounted on one glass slide and imaged together. Gain was adjusted for each slide at the saturation limit of the root showing maximum glow and then set for all the roots on the same slide. To identify any spatial differences in ROS accumulation in each root, we took multiple high magnification fluorescent images along the longitudinal axis of root and stitched them into one complete image. This stitched image was then quantified in five different developmental zones: 4 equal length zones between root tip and first visible root hair and the last one as root hair differentiation zone. Mean fluorescent value for each zone was calculated in FIJI. Two biological replicates were performed with 4 seedlings per replicate and 4-5 seminal root tips per seedling were analysed. Statistical analysis was performed using Welch's t-test in "RStudio". *, **, *** indicate significant *P*-value < 0.05, 0.01, 0.001 (n roots=16-20, n plants=4, n experiments=2).

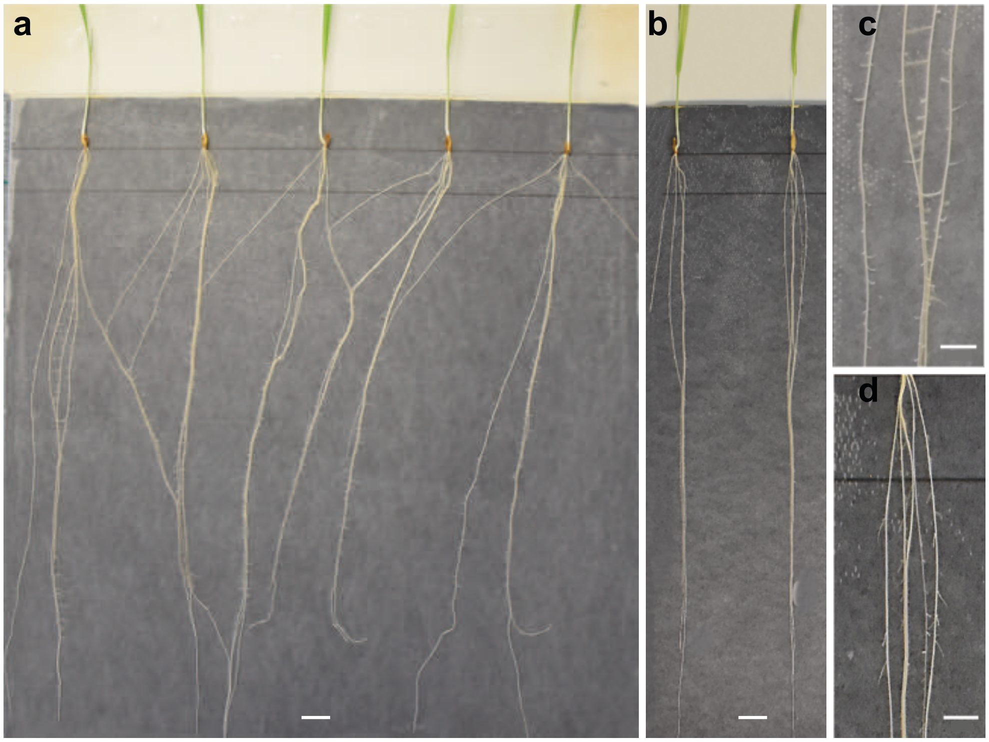

Fig. S1. TM194 mutant shows steeper seminal and lateral root angle phenotype in semi-hydroponic conditions.
a-b, 2D Root architecture phenotype of 10 DAG seedlings of barley **a**, cv. Morex. and **b**, TM194 in semi-hydroponic conditions. Scale bar = 1cm. **c-d**, Magnified image of **c**, Morex and **d**, TM194 seminal roots highlighting phenotypic difference in lateral root insertion angles. Scale bar = 1 cm.

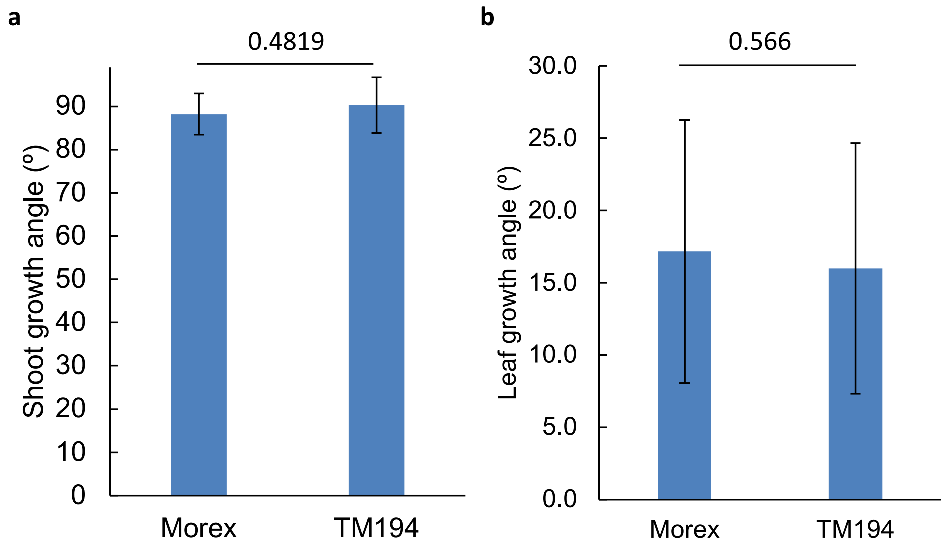

Fig. S2. TM194 mutant do not show significant difference in shoot growth angle at seedling stage and leaf growth angle at flowering stage.

a, Bar plot showing mean +/- standard deviation of coleoptile growth angle in 8 individual seedlings of mutant and Morex during seedling stage (7 days) grown vertically in a growth pouch system. No statistical difference (Student's *T*-test, P-value 0.4819) in growth shoot growth angle was observed for mutant vs Morex. **b**, Bar plot showing +/ standard deviation of leaf growth angle in three young leaves in TM194 mutant and Morex during flowering time. Three leaves each were measured from > 13 individual plants grown in pots (TM194, n=39 leaves and Morex, n=42 leaves) and Welch's test was used to assess statistical difference (Not significant, *P*-value 0.566). Both Morex and TM194 mutant showed similar flowering time.

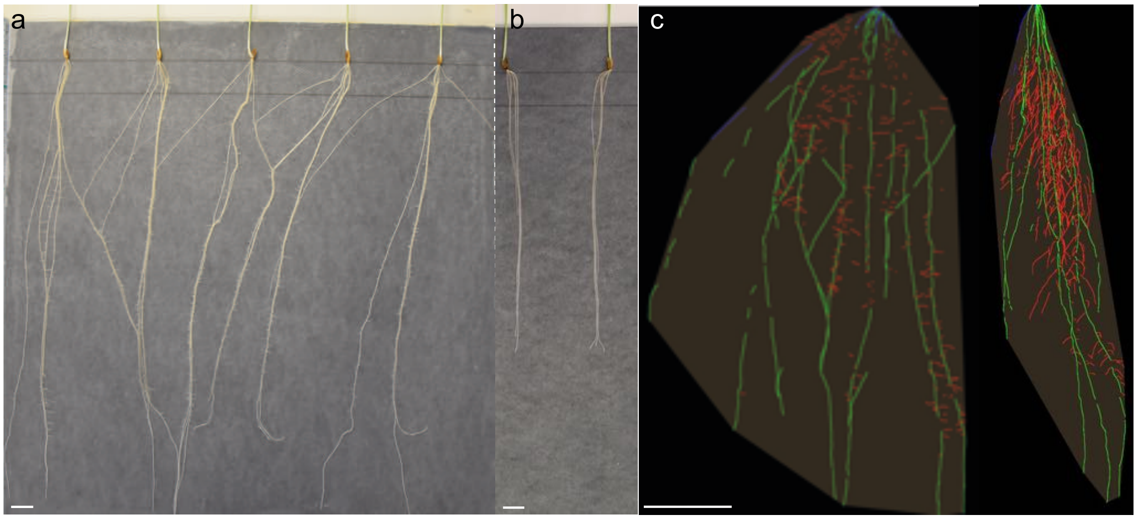

Fig. S3. TM3580 mutant shows steeper seminal and lateral root angle phenotype in semi-hydroponic conditions.
a-b, 2D Root architecture phenotype of 10 DAG seedlings of barley **a**, cv. Morex. and **b**, TM3580 in semi-hydroponic conditions. Scale bar = 1cm. **c**, Representative image of 20 DAG Morex and TM3580 revealing difference in lateral root insertion angles (red coloured) grown in soil-filled rhizotrons. Scale bar = 10 cm.

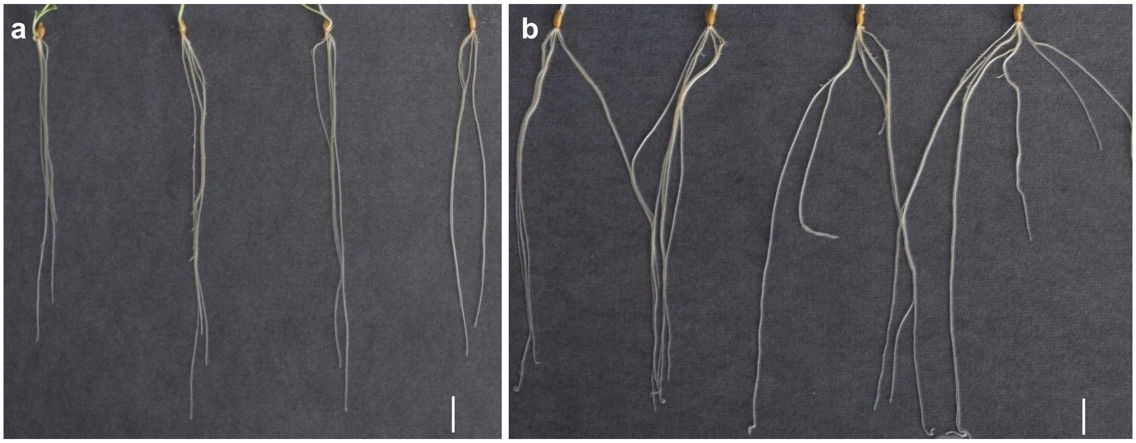

Fig. S4: TM194 and TM3580 barley enhanced gravitropic mutant lines do not complement and therefore highlight the same mutant locus

a, 2D root architecture of progenies derived from the cross TM194 × TM3580 (F1 cross), seven days after germination, in semi-hydroponics condition. **b**, 2D root architecture of cv. Morex (wildtype) grown as control, same conditions. Scale bar = 2cm.

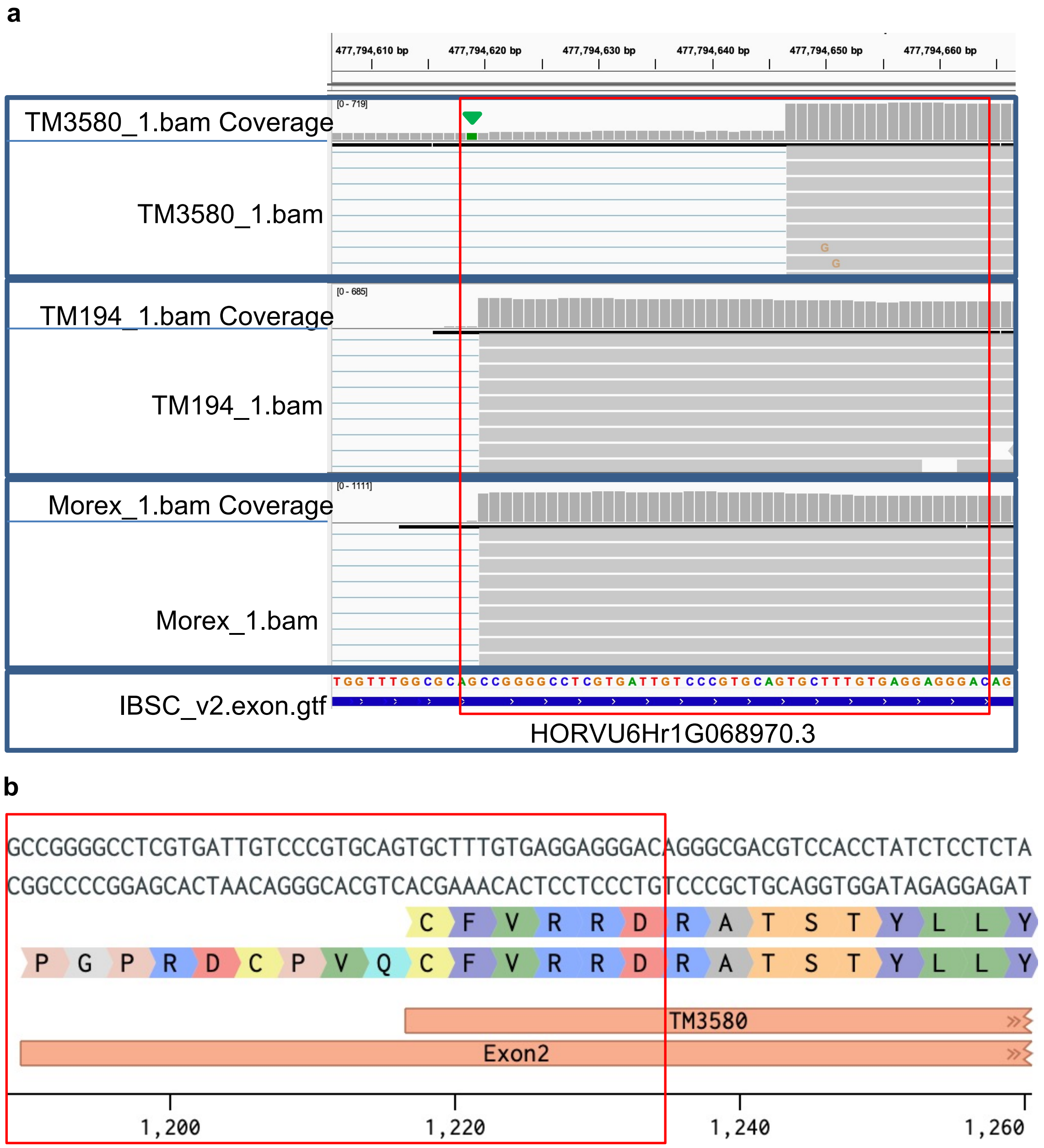

Fig. S5. TM3580 mutation causes a splice acceptor variant, resulting into a deletion of 9 amino acids without any frameshift.

a, Snapshot of Integrative Genomics Viewer (7) showing visualisation of G to A splice acceptor mutation in TM3580 (nucleotide position 'G' > A' is marked by green arrow) causing trimmed version of Exon2 in TM3580 (compared to Morex and TM194) mapped to barley reference genome (IBSC_v2.exon.gtf). **b**, Schematic showing that the mutation in TM3580 causes 9 amino acid deletion without any frameshift. Red box in **a** and **b** highlights starting region of Exon2 in Morex and TM3580.

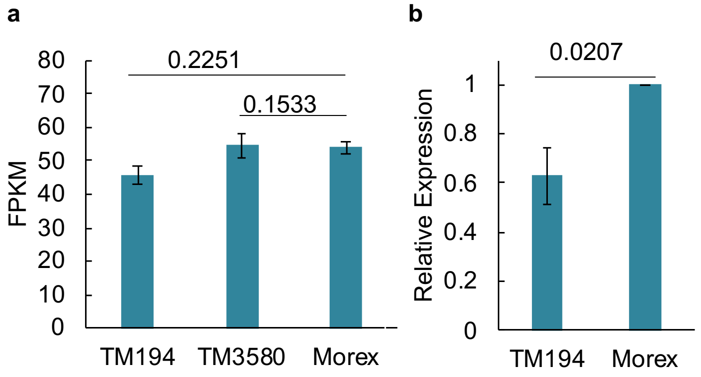

Fig. S6. Expression of *HvEGT1* in mutant alleles compared to Morex.
a, Bar graph showing number of *HvEGT1* transcripts (FPKM, Fragments Per Kilobase of transcript per Million mapped reads) measured in Morex and *Hvegt1* mutant alleles (TM194 and TM3580) root tips in our RNAseq dataset. Benjamini-Hochberg FDR corrected *P-values* are shown for comparison between Morex vs TM194 and TM3580 vs Morex. **b**, Bar graph showing relative expression of *HvEGT1* measured in Morex and TM194 root tips using RTqPCR. Statistical significance *P*-value calculated using Student's *T*-test, is shown.

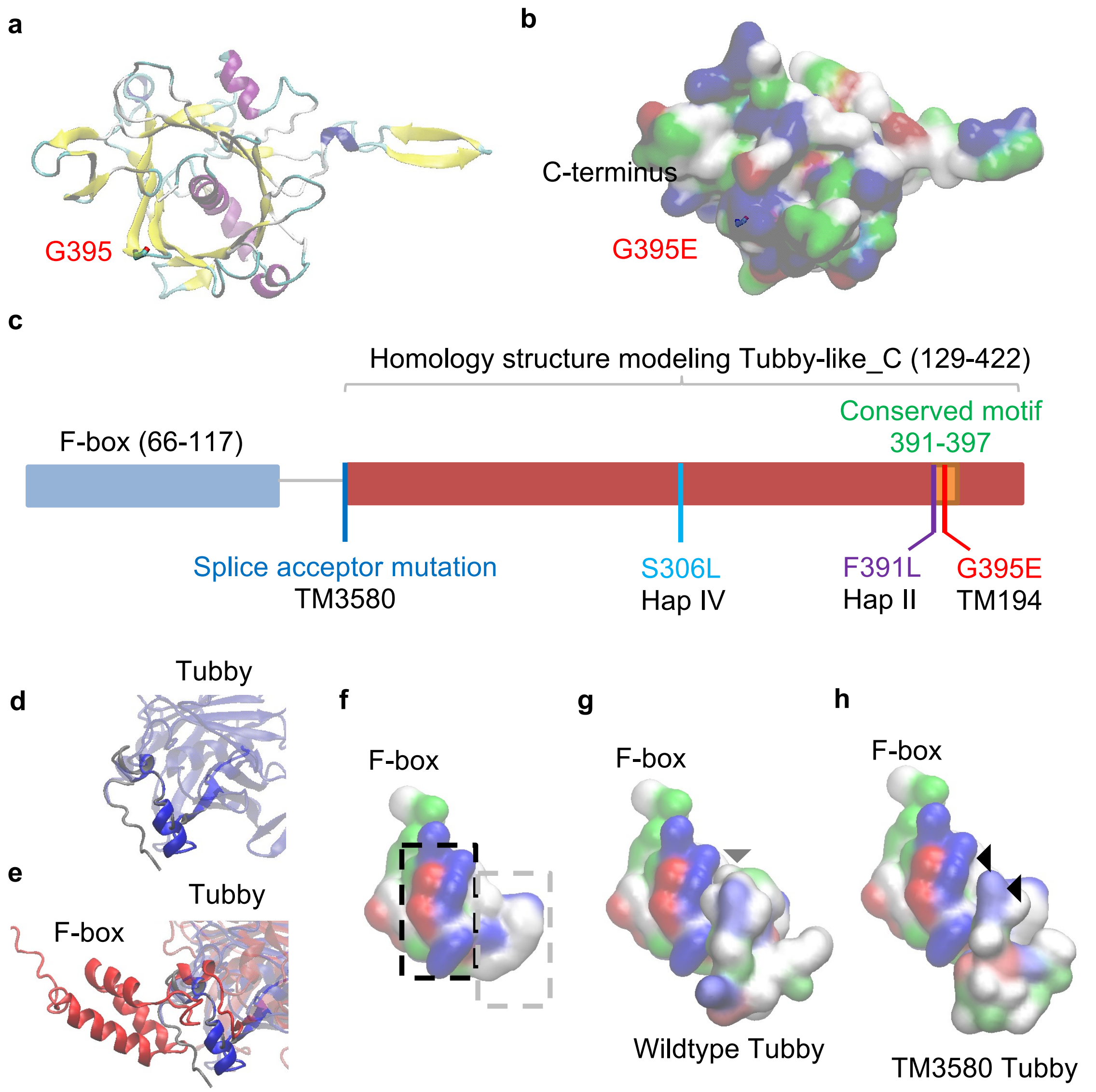

Fig. S7. Mapping of mutant alleles and key haplotypes identified from the WHEALBI population onto the HvEGT1 Tubby domain structure.

a, Homology model for the Tubby-like domain of HvEGT1 obtained using Phyre2 (8). Magenta, yellow, cyan and white color indicate alpha helices, beta strands, turns and random coils, respectively. G395 indicates the mis-sense substitution position of TM194 mutant on the predicted domain. G395 sits in a highly positively charged cavity, probably stabilized by an adjacent negatively charged C-terminal site. G395E substitution leads to a small, neutral amino acid changing to a larger, negatively charged residue that is highly likely to destabilise this region of the protein, likely affecting its function. **b**, Amino acid charge distribution in **a**. **c**, Domain organisation and mapping of TM3580 and TM194 mutations and haplotypes II and IV identified from the WHEALBI (9) population. **d,** Superposition of wildtype (gray) and TM3580 (blue) Tubby domains indicates presence of a new alpha helical segment (solid blue) in the N-Terminal region. **e,** Alphafold2 prediction of full EGT1 protein (red) shows that the structural changes of TM3580 (blue) are juxtaposed with the interaction interface of the Tubby and F-box domains (red). **f,** Interaction interface of F-box domain is part charged (boxed black), part charged hydrophobic (boxed grey) and is complemented by the wildtype Tubby domain interface (grey arrowhead) **g.** **h,** TM3580 mutant changes brings two negatively charged residues together on the interface (black arrow heads), likely destabilising structure and function of EGT1. All surface representations are color coded as follows - blue (basic, positively charged), red (acidic, negatively charged), green (polar) and white (non-polar) residues.

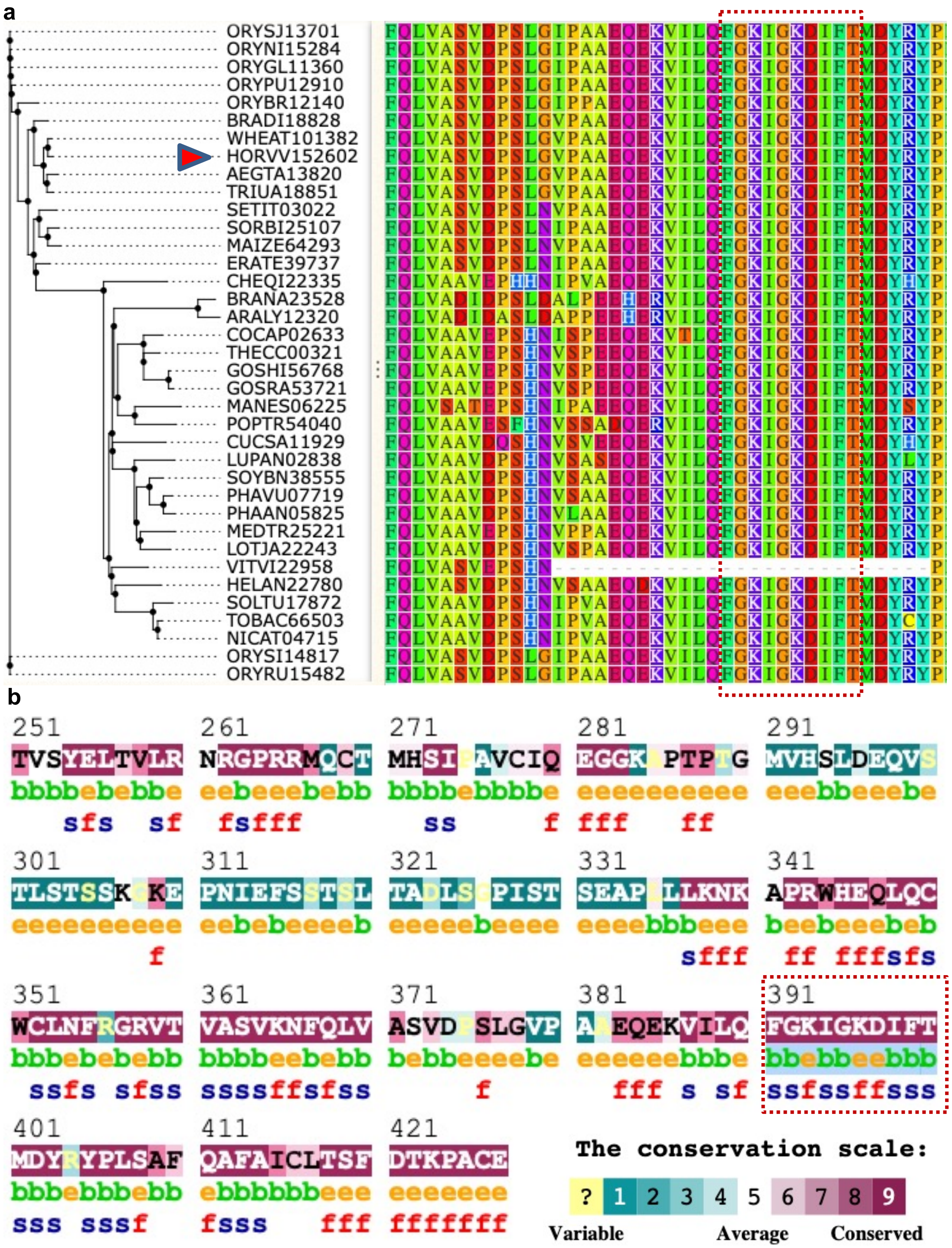

Fig. S8. Phylogenetic conservation of HvEGT1 residues.

a,Trimmed multiple sequence alignment and phylogenetic tree using WASABI (http://wasabiapp.org, through ConSurf (10)) showing conserved residues 391 to 400 (red dotted rectangle) in the homologous sequences of HvEGT1 from 37 other plant species (UniProtKB - F2DEK2_HORVV; Phylogenetic database OMA (11) Group 512684). Red filled arrow indicates HvEGT1. **b**, Conservation to function prediction using ConSurf (12) highlighting residues 391 to 400 (red dotted rectangle). G395E and haplotype II (Fig. 4d-e) lie within this highly conserved region. Colored letters indicate, 'e' = exposed and 'b' = buried residues according to the neural-network algorithm; 'f' = predicted functional residue (highly conserved and exposed) and s = predicted structural residue (highly conserved and buried).

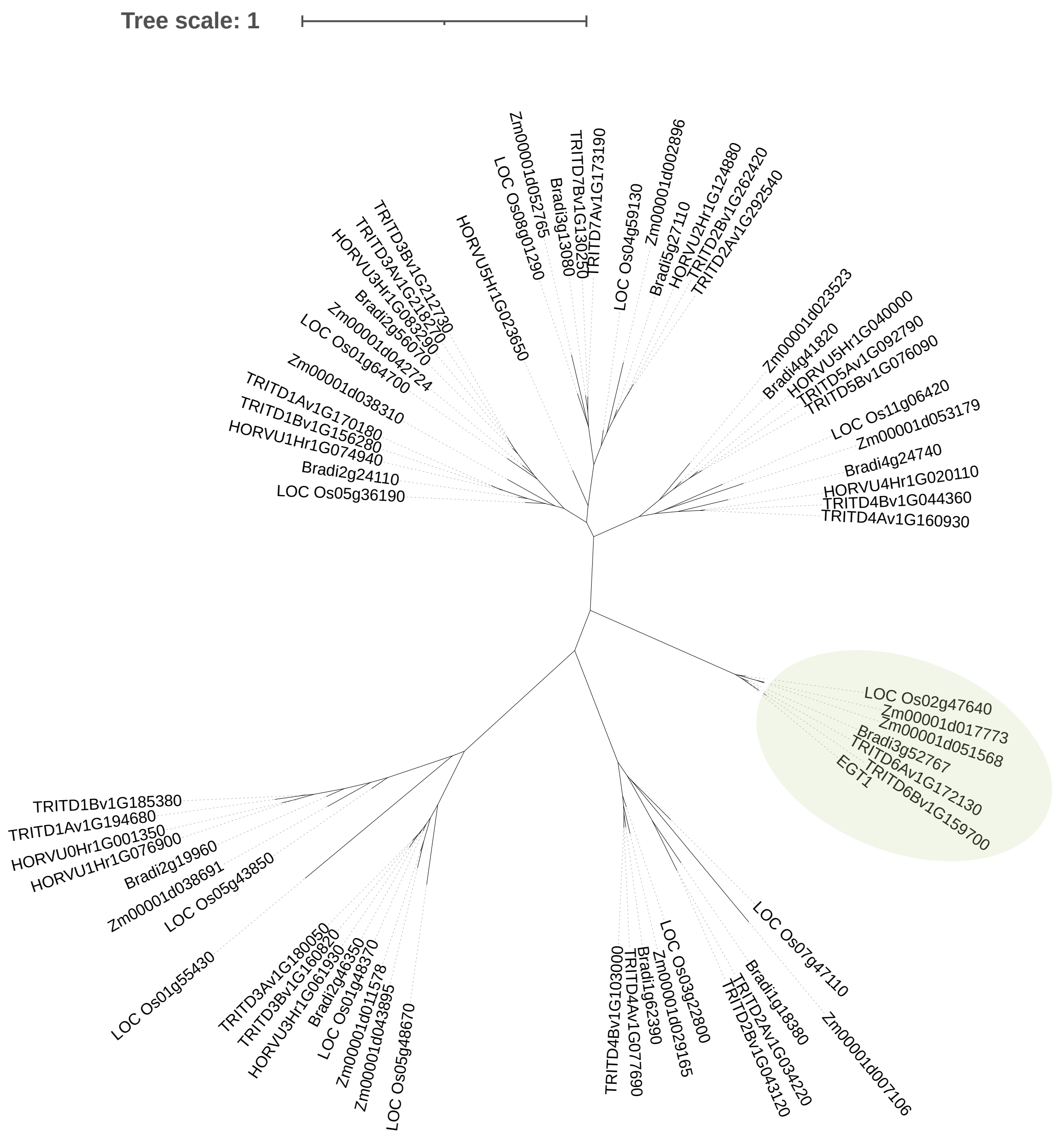

Fig. S9. Phylogenetic analysis of Tubby-like F-Box Protein sequences in barley, wheat, rice, maize and brachypodium.
HvEGT1 was used as a seed gene to select orthologous genes (>40% identity) from key monocot species such as barley (*Hordeum Vulgare*), wheat (*Triticum turgidum*), rice (*Oryza Sativa spp. Japonica*), maize (*Zea Mays B73*) and brachypodium (*Brachypodium distachyon*) using interactive phylogenetic module of Monocots Plaza 4.5 (3). Protein sequences were aligned using MUSCLE (13) and tree was constructed using FastTree (14) algorithm. Generated Newick file was imported into iTOL(15) to create an unrooted tree. Light green color highlights the distinct clade formed by *HvEGT1* with 2 orthologs each from wheat and maize and 1 ortholog each from rice and brachypodium.

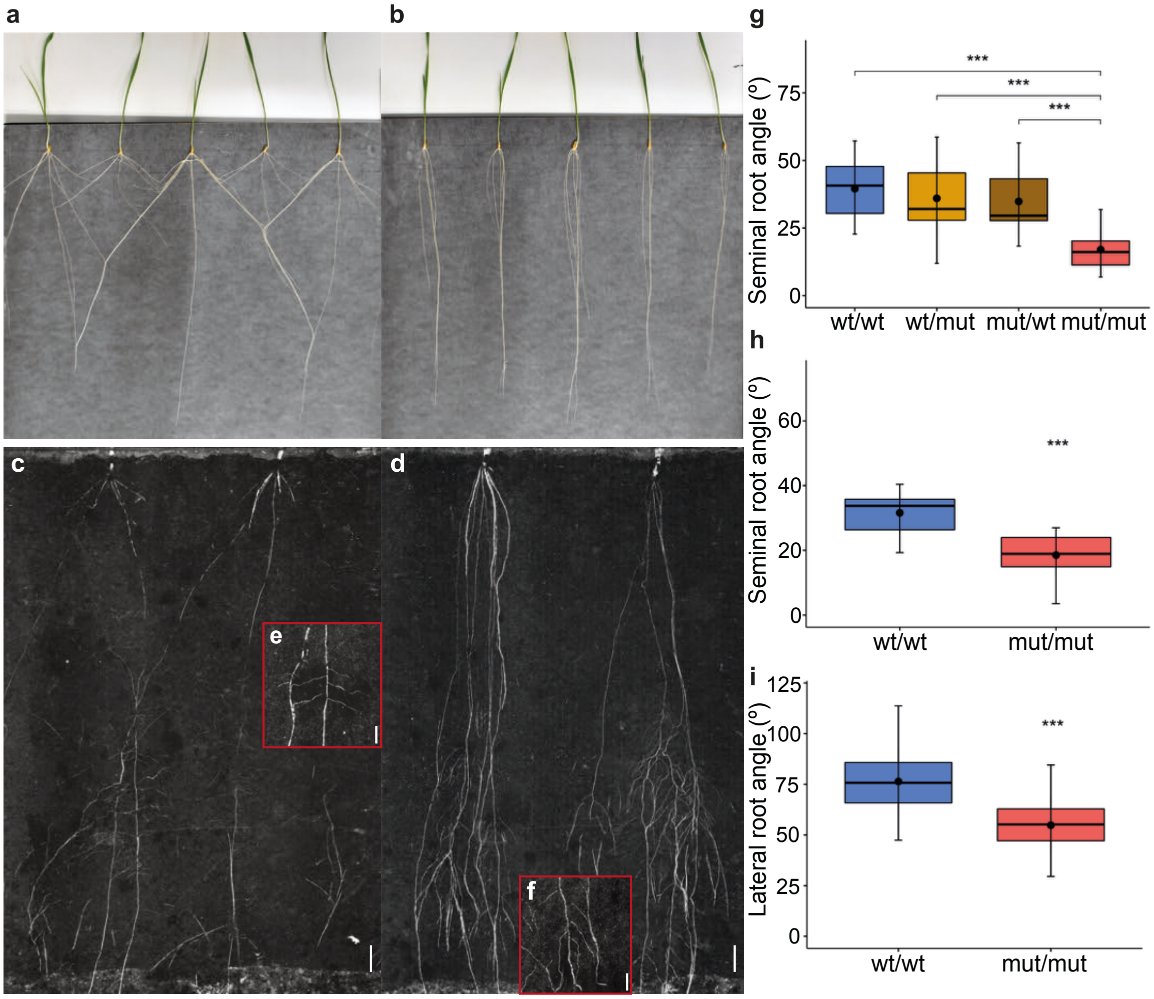

Fig. S10. *TdEGT1* loci also control root growth angle in wheat.

a-b, Representative images showing root growth angle 8-days old a, wildtype *TdEGT1* (wtA/wtB) and **b**, homozygous cross of *Tdegt1* mutant (mutA/mutB) grown in semi-hydroponic condition. Scale bar = 2 cm. **c-d**, Representative images of 20-days old **c**, wtA/wtB and **d**, mutA/mutB mutant grown in soil rhizotrons. Scale bar = 2 cm. **e-f**, Representative magnified images of lateral roots from **c** & **d**, respectively. Scale bar = 1 cm. **g**, Quantification of seminal root angle from 8-days old wtA/wtB, wtA/mutB, mutA/wtB and mutA/mutB grown in semi-hydroponic condition. **h**, Quantification of seminal root angle from 12-days old wtA/wtB and mutA/mutB grown in soil rhizotrons. **i**, Quantification of lateral root angle (from seminal roots) of 20-days old wtA/wtB and mutA/mutB grown in soil rhizotrons. *** shows statistically significant difference between wild types at one or both homologs (wtA/wtB, mutA/wtB and wtA/mutB) versus double mutants (mutA/mutB), assessed using Welch's t-test, $p<0.001$, n=12-28 seminal roots and 86-91 lateral roots.

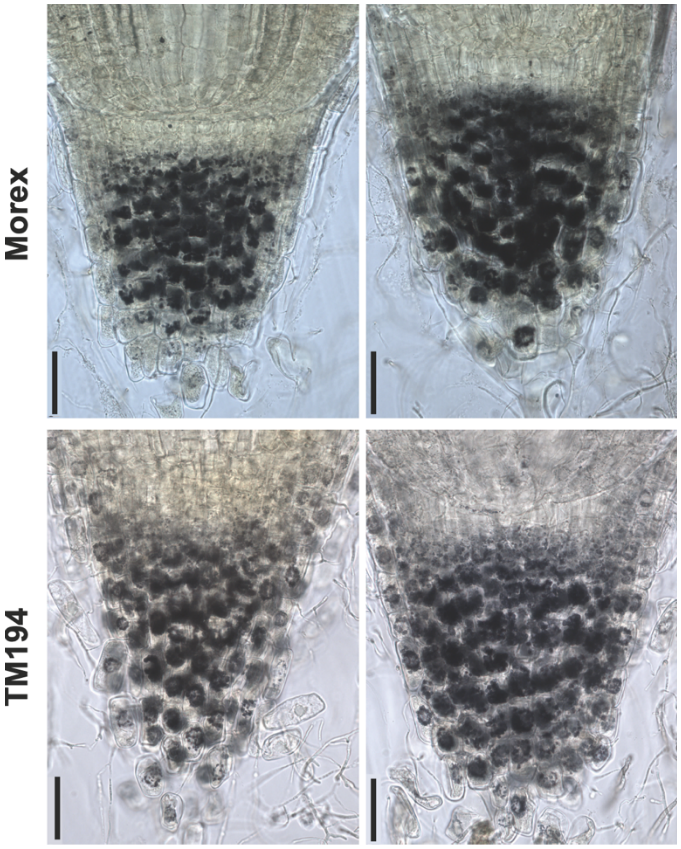

Fig. S11: Lugol staining showing no difference in statolith-starch granule levels in *hvegt1* (TM194) mutant root tips compared to Wildtype (cv. Morex).
Vibratome sectioning images showing 5-day old seminal root tips of TM194 mutant and Morex stained with Lugol's iodide solution. Two representative images per genotype are shown. Scale bar = 100 µM.

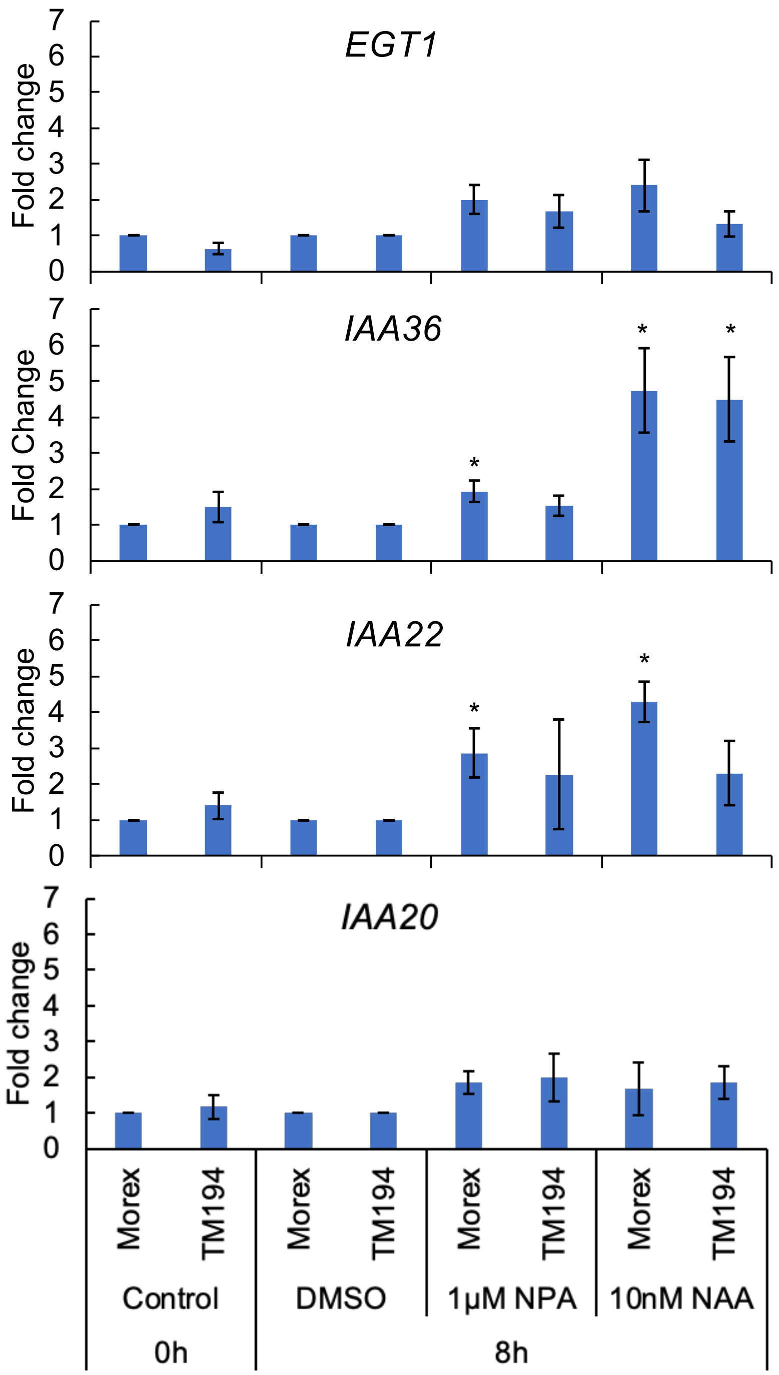

Fig. S12: RTqPCR results showing *HvEGT1* expression after NAA and NPA treatments. Bar graph showing RTqPCR analysis of *HvEGT1*, *HvIAA36, HvIAA22* and *HvIAA20* expression in TM194 mutant and Morex measured under control, DMSO control,10nM NAA and 1uM NPA treatments. Here, control represents experimental conditions where plants were grown in 1% Agar plates supplemented with ½ Hoagland's solution and grown in 16/8h photoperiod and a temperature 22ºC/18ºC for 3 days prior transferring to treatment on 1% Agar plates supplemented with ½ Hoagland's solution, 0.1% DMSO and either 10nM NAA or 1µ M NPA. 5mm seminal root tips from >3 plants (i.e., ~15 root tips) were pooled for RNA extraction and 3 independent biological replicates were performed. Data shows mean +/- standard error fold change. *T*-test was performed to assess the statistical difference. * *indicates P-value < 0.05* and 1.5 >=Fold change<= -1.5 between treated vs DMSO samples for each genotype.

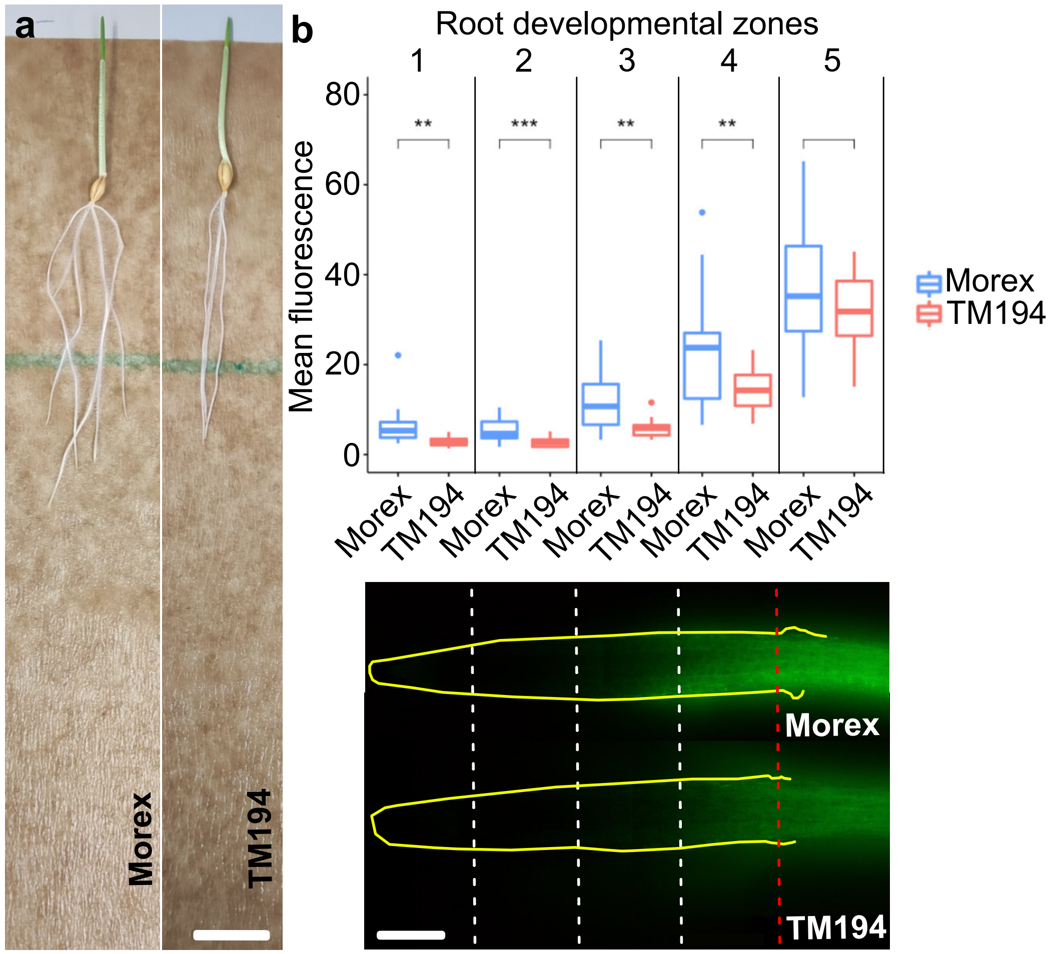

Fig. S13: *Hvegt1* (TM194) shows enhanced levels of ROS in root tips of mutant versus wildtype (cv. Morex).

a, Representative images of four days old seedlings of wildtype Morex and TM194 mutant. Only seedlings with equal root growth were used for ROS detection assay. Scale bar = 2cm. **b,** Boxplot (top) showing quantified mean fluorescence in different developmental stages of seminal roots stained with 30 µM CM-H_2DCFDA (see SI Appendix). Example of fluorescent image (bottom) of Morex and Hvegt1 mutant allele (TM194) used for quantification. Scale bar = 500µm. Yellow outline marks the root. Each fluorescent image was virtually sectioned into five root developmental zones (1-4 equal length root tip growth regions prior to first visible root hair and 5th root hair differentiation region). Experiment was replicated twice with 4 seedlings per replicate and 3-4 seminal roots per seedling (i.e., N = 27-35 roots) were analysed.

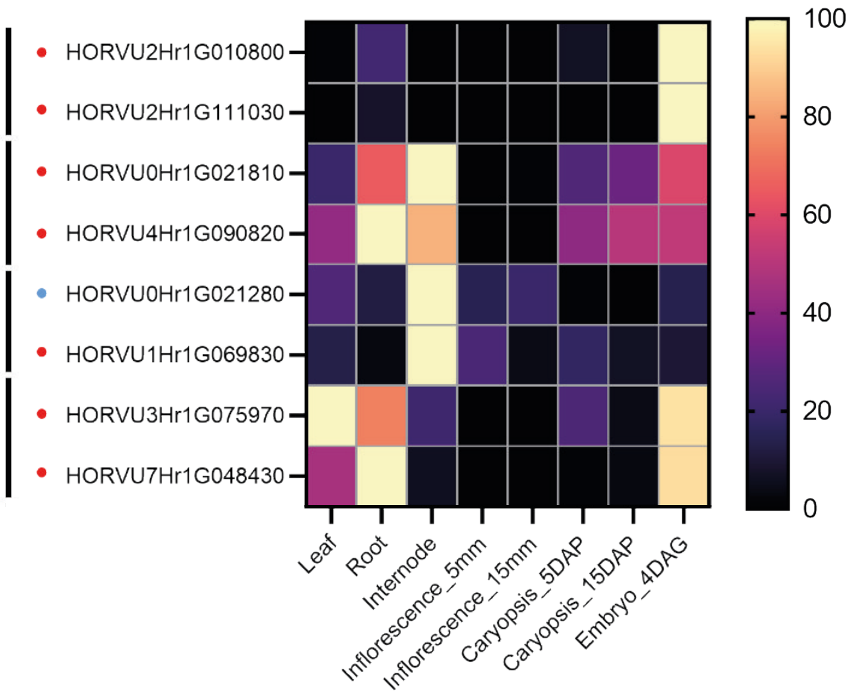

Fig. S14. Cell wall gene expression clusters that likely to respond to loss of *HvEGT1* expression.

Heat map shows relative transcript abundance of cell wall related genes differentially expressed in *Hvegt1* mutant alleles (TM194 and TM3580), in a previously published barley RNAseq dataset (16). Expression values (RPKM) were transformed to proportions of the maximum expression value observed for each gene. Red dots indicate the genes are down-regulated in *Hvegt1* mutant alleles; blue indicates up-regulation. Bars indicate different modules. The changes in expression suggest a complex deregulation of the cell wall machinery in the root of *Hvegt1* mutant alleles. For instance, *HORVU2Hr1G010800* (EXP) and *HORVU2Hr1G111030* (XET) are co-expressed in the root and germinated embryo, and both are downregulated in Hv*egt1*.

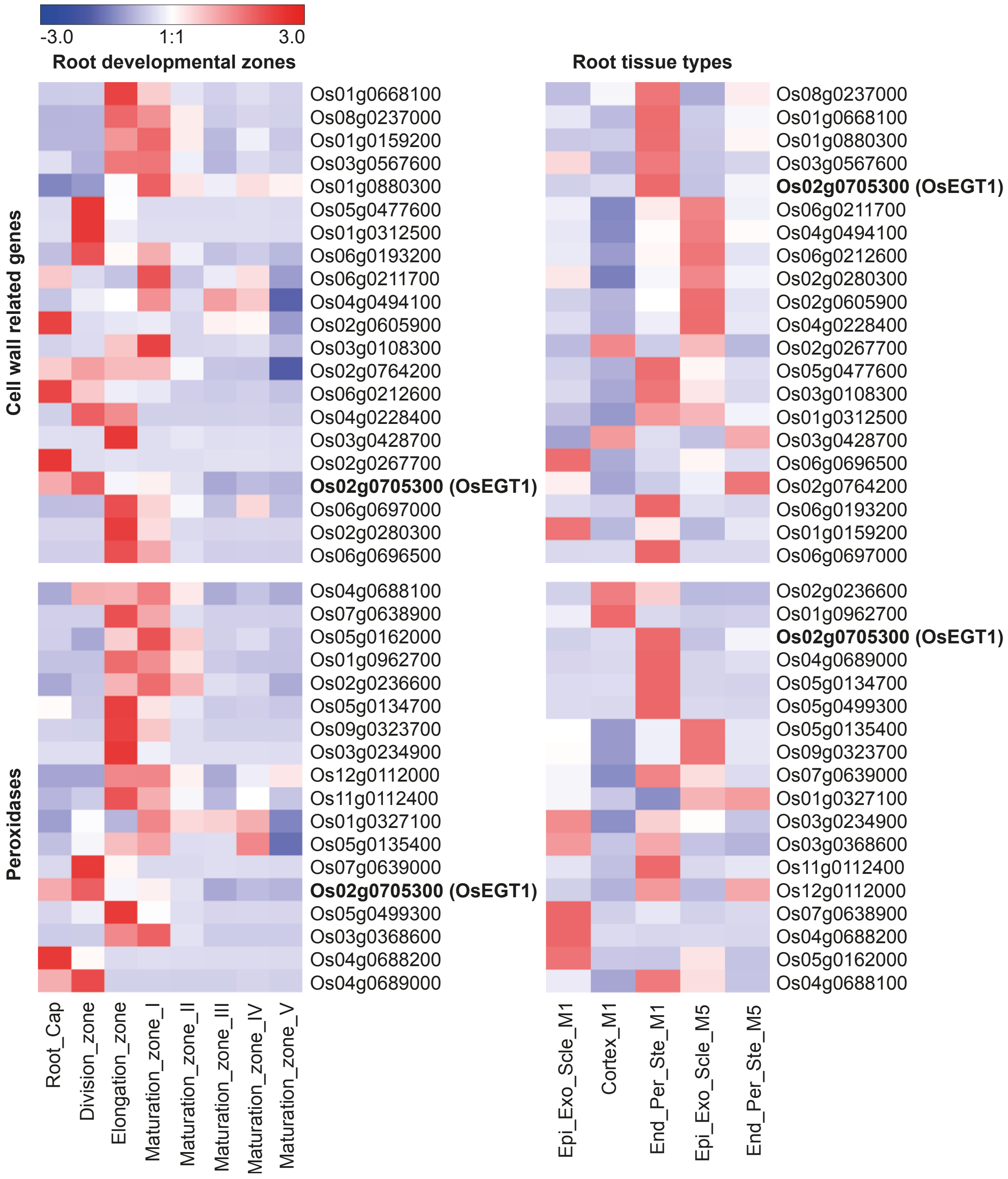

Fig. S15. Spatiotemporal expression of selected cell-wall and peroxidases related genes in rice roots.
Heat map shows relative abundance of cell-wall and peroxidases related genes, in rice root developmental stages and tissue types (17), orthologous to genes that are differentially expressed between Morex and *Hvegt1* alleles. Plaza 4.5 (3) monocot comparative genomics database was first used to identify best hit family orthologs. Next, Rice Xpro 3.0 (18) was used to extract mean raw Cy3 signal intensities for non-overlapping orthologs from RXP_4001 transcriptome experiment. Heatmap.2 package in R-studio was used to generate heatmaps and WPGMA method was used to create Hierarchical clustering of genes (clustering tree was trimmed to accommodate all images into this figure). Epi.Exo.Scle. = Epidermis/Exodermis/Sclerenchyma; End.Per.Ste. = Endodermis/Pericycle/Stele, M1 = Maturation zone_I, 1-3mm from root tip; M5= Maturation zone_V, 10-20mm from basal side. Gene id in bold indicates rice ortholog of *HvEGT1*.

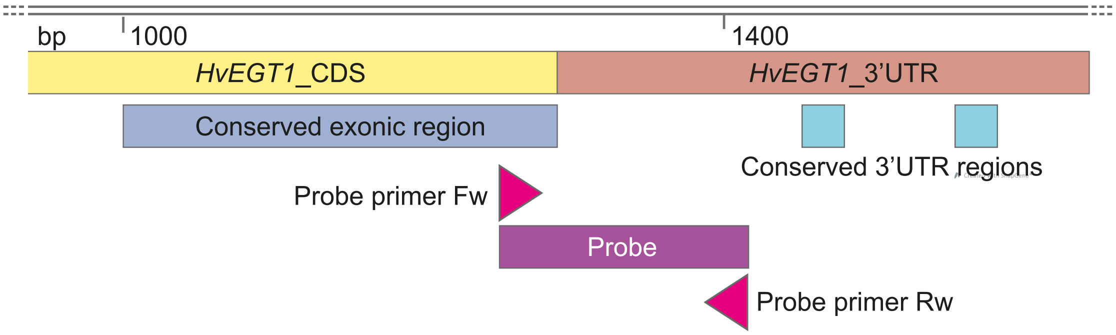

Probe Sequence (156bp):
CTAGCTTCGACACCAAACCGGCCTGCGAATGACCGAAGAAGGCGGCGTTAACCGGGC
CAGCCAGCTTGAGACATTACCTGCCCCTTGGATGGTGTAAATTCTGATATCTTTGCTTCA
ATTCTCGGACGCTGCCCCTGGGGAGATGGATGGTAGGTG

Fig. S16. Schematic of probe design for *EGT1* RNA in situ hybridisation experiment. The complete coding sequence and 3'UTR sequence of *HvEGT1* (HORVU6Hr1G068970) were obtained to design probes (top part). The three conserved regions were annotated as shown in the diagram, including one at the end of coding sequence (998-1284bp), and two in the 3'UTR region (1428-1438bp and 1529-1539bp). The primers were designed to frame partial coding sequence and partial 3'UTR and avoided conserved sequence to increase the specificity of probe recognition. To syntheses the antisense and sense probes, an adaptor sequence (T7 promoter, TAATACGACTCACTATAGGG) was added to the reverse and forward primer, respectively. The probe (bottom part) used in this study is 156bp.

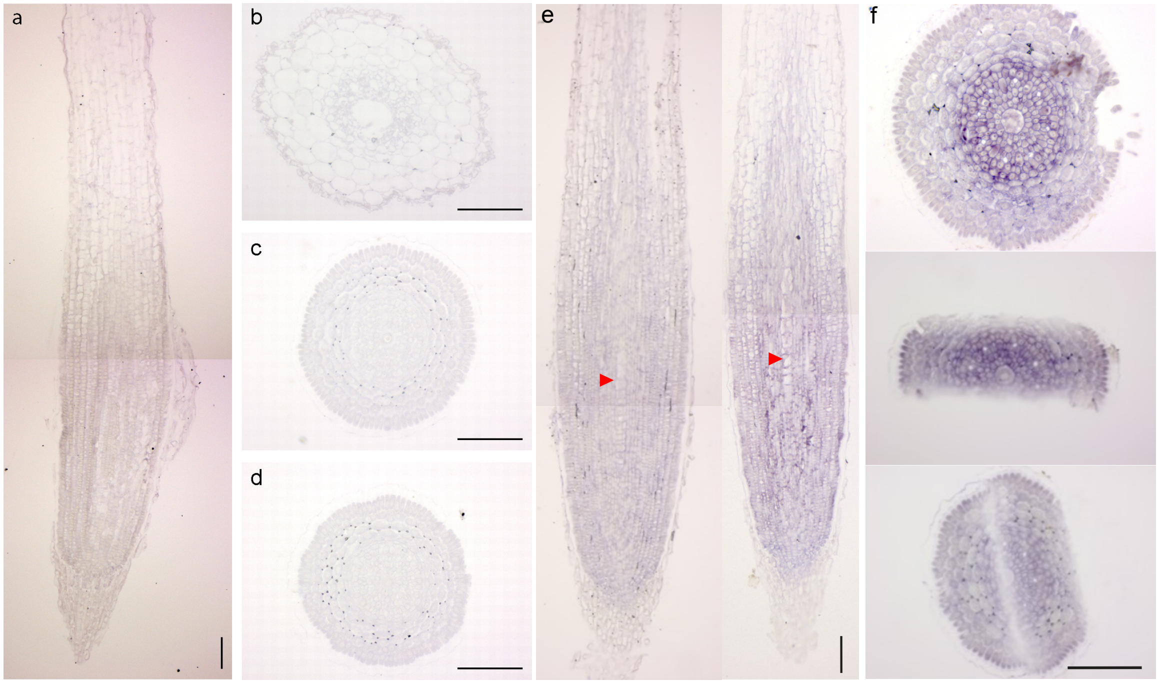

Fig. S17. ***In Situ*** **Hybridisation (ISH) on root tips of Morex with** ***HvEGT1*** **sense and anti-probe.**

a, ISH using sense probe on longitudinal section of root tip of Morex. **b-d**, ISH using sense probe on cross-sections of Morex in elongation-division zone (**b**), higher proximal meristem (**c**) and lower proximal division (**d**) zones. **e-f**, additional replicates of ISH (of Fig4c & 4e respectively) using *HvEGT1* anti-sense probe on longitudinal (**e**) and cross sections (**f**) of Morex root tip. Red arrow heads on (e) show exposed central metaxylem, suggesting roots were sectioned at the center of the root. Scale bar = 100µm.

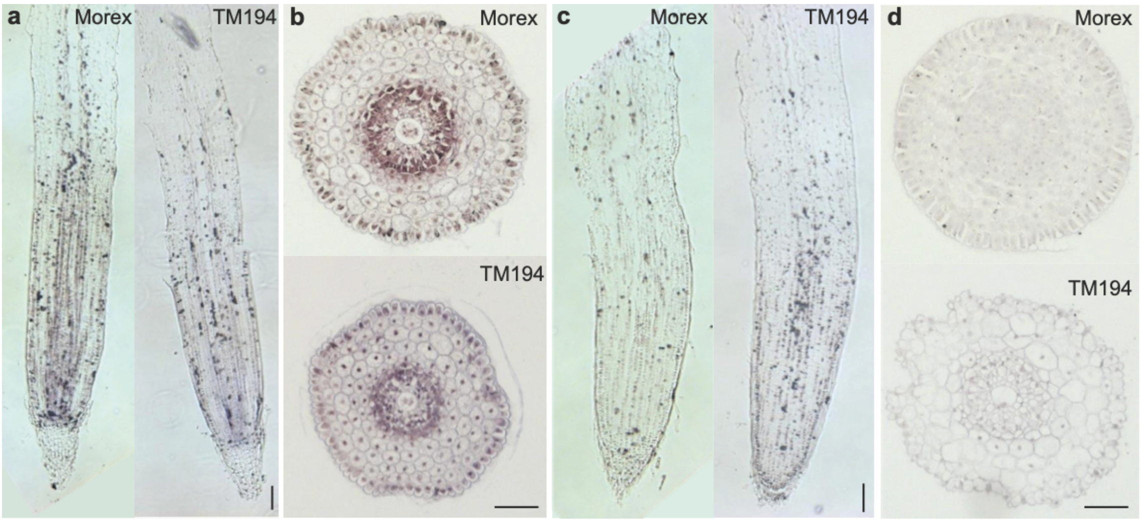

Fig. S18. *In Situ* Hybridisation (ISH) on root tips of TM194 and Morex with *HvEGT1* anti-sense and sense probes.
a-**d**, ISH using *HvEGT1* anti-sense probe (**a-b**) and sense probe (**c-d**) on longitudinal section (**a,** **c**) and on cross-sections (**b** and **d**) of Morex and TM194 in the elongation zone. Scale bar = 100 µm.

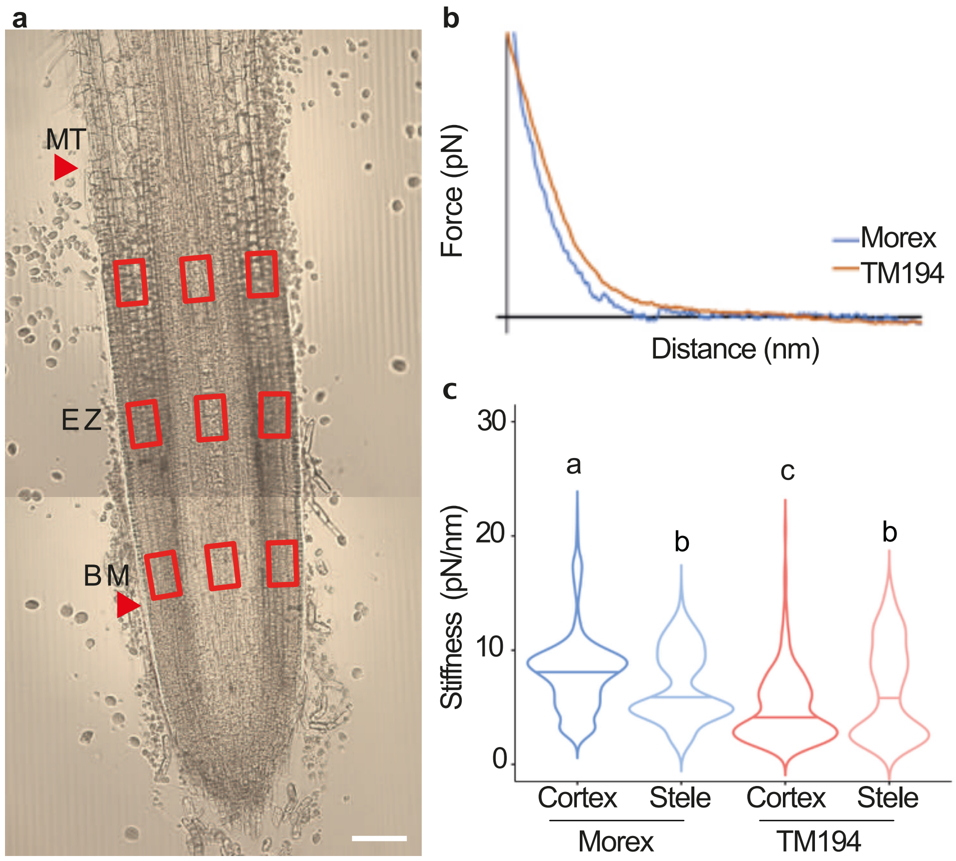

Fig. S19: AFM nanomechanical force spectroscopy of tissues within the elongation zone of the root meristem.
a, Example of a 50 µm longitudinal section of Morex (MT – maturation zone, EZ – elongation zone and BM – basal meristem). Red rectangle show approximate locations of AFM force spectroscopy tests. Indentations were performed in the observable centre of root meristem cells on each section generating a total of 100 < n < 360 force curves for each biological replicate. **b**, Example showing typical force-distance curves measured on Morex and TM194 sections. The slope of the indentation part of the curve was used for determining apparent stiffness (pN/nm). **c**, Summary of force spectroscopy results showing the stiffness values of the stele and cortical tissues of the root. The pooled data from each area was analysed using a non-parametric Wilcoxon test for significant differences between sample type and area ($p < 0.001$).

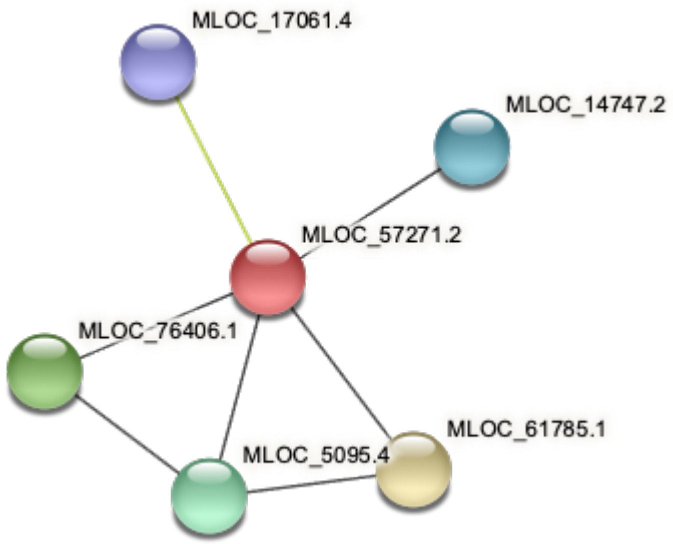

Fig. S20: Protein-protein interaction database analysis suggests that EGT1 regulate cell elongation and cellulose synthesis related proteins.
The protein-protein interactions (experimental, text mining, database curated and co-expression) of *HVEGT1* were studied using String Database (https://string-db.org) to identify five high-confidence interactions (4 co-expression – black colored edges and 1 database curated – green colored edge). MLOC_14747.2 (*HORVU2Hr1G032710*) and MLOC_61785.1 (*HORVU2Hr1G063820*) are orthologous to *Arabidopsis thaliana* LONGIFOLIA3, one of the 4 proteins that control polar cell elongation by regulating cell wall modifying enzymes encoded by a multi-gene family xyloglucan endotransglucosylase/hydrolase (19). Quadruple mutant of LNG family members have shown to have reduced cell elongation and reduced organ sizes. MLOC_76406.1 (*HORVU7Hr1G061070*) is orthologous to *Arabidopsis thaliana* SHOU4, a plasma-membrane-localised proteins that negatively regulate cellulose synthesis by inhibiting the exocytosis of CESAs (cellulose synthases) (20). SHOU4 mutant cells in inflorescence stem are shown to be smaller and with thinner cell walls. MLOC_5095.4 (*HORVU5Hr1G035610*) is orthologous to KIPK (KCBP-Interacting Protein Kinase) speculated to be involved in regulation of cell expansion through transport of cell wall material (21). MLOC_17061.4 (*HORVU7Hr1G097840*) is orthologous to *Arabidopsis thaliana* a plant-specific GATA-type transcription factor family protein.

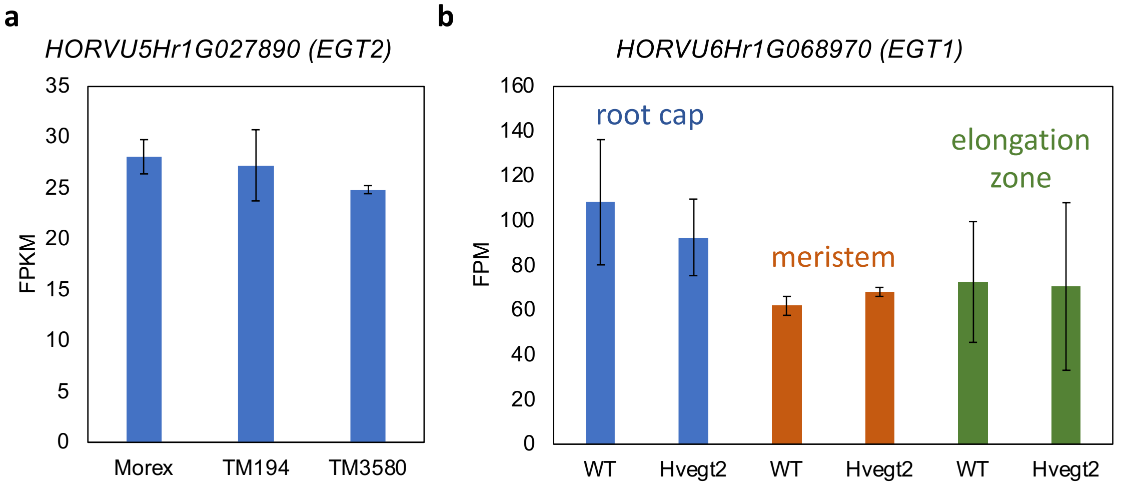

Fig. S21: EGT1 and EGT2 could function in parallel AGO pathways to control root angle in barley.

Bar graphs represent **a,** measured expression (FPKM values) of *EGT2* in Morex and egt1 mutant alleles (TM194 and TM3580) and **b,** measured expression (FPM values) of *EGT1* in wild type and *Hvegt2* mutant in different root growth zones (root cap, meristem and elongation zone) (22). These graphs suggest that expression of *EGT1* in *egt2* mutant largely remain unchanged and vice versa.

Supplementary Table 1. Primers used for qPCR analysis in this study.
HvAlpha-Tub (*HORVU1Hr1G081280*) and *HvGAPDH* (*HORVU6Hr1G054520*) were used as internal control. Auxin responsive gene *HvIAA36* (HORVU0Hr1G021630.1), *HvIAA22* and *HvIAA20* primers used from Shi et al. (4).

Primer	Gene ID	Sequence
Alpha_TUB_F	HORVU1Hr1G081280.1	AGTGTCCTGTCCACCCACTC
Alpha_TUB_R	HORVU1Hr1G081280.1	AGCATGAAGTGGATCCTTGG
GAPDH_F	HORVU6Hr1G054520.3	GGAGGAGTCTGAGGGAAACC
GAPDH_R	HORVU6Hr1G054520.3	GCTGTATCCCCACTCGTTGT
EGT1_F	HORVU6Hr1G068970.3	GCTTGCAGCCCGCAAGATCA
EGT1_F	HORVU6Hr1G068970.3	ACCGCAGCATCACAAGGAGG
IAA36_F	HORVU0Hr1G021630.1	GCTGGAGCTTCTGTCCAAG
IAA36_R	HORVU0Hr1G021630.1	TCTTCCTGTAACTGCGGATTG
IAA22_F	HORVU5Hr1G093580.3	CCACCTACGAGGACAAGGA
IAA22_R	HORVU5Hr1G093580.3	CCTGCTCTTGCATTTCTCCA
IAA20_F	HORVU5Hr1G014300.1	AGCTGATGGATCTGCTCAAC
IAA20_R	HORVU5Hr1G014300.1	GGCCAATAGCTTCTGATCCTT

SI Datasets

Dataset S1. Exome sequencing results of TM194 and TM3580 compared against Morex highlighting (red) mutations within *HORVU6Hr1G068970* (*HvEGT1*), encoding Tubby-Like F-box domain protein.

Dataset S2. Transcription factor binding site prediction of 2.5kb promoter of *HvEGT1* analysed using PlantRegMap tool (5) (http://plantregmap.gao-lab.org/binding_site_prediction.php). The promoter sequence is provided in the second worksheet.

Dataset S3. Differentially expressed genes between comparisons of TM194 vs Morex, TM3580 vs Morex and TM194 vs TM3580. Genes with Benjamini Hochberg controlled FDR < 0.05, FC +/- 1.5 and FPKM >1 were assigned as differentially expressed.

Dataset S4. gProfiler GO enrichment analysis of selected 841 genes that are differentially expressed in *Hvegt1* mutant alleles compared to Morex. Green filled section indicates biological processes (BP) used for further analysis.

Dataset S5: Auxin Transport (GO:0060918) and Auxin Biosynthesis (GO:0009851) genes differentially expressed in TM194 vs Morex, TM3580 vs Morex and TM194 vs TM3580 comparisons in our RNAseq dataset. Arabidopsis genes annotated to these parent and child GO terms were used to identify orthologous genes in Barley using gProfiler's gOrth tool (6). The second and third worksheets included gOrth results. Green filled rows indicate genes that are simultaneously differentially expressed in both the mutant alleles compared to wildtype Morex.

Dataset S6. List of genes enriched in hydrogen peroxide metabolic and cell wall organisation or biogenesis processes. Orthologous genes in Arabidopsis and rice were identified using Plaza 4.5 (3) comparative genomics database and only BHIF (Best-Hits-and-Inparalogs(BHI)family) evidence was retained.

SI References

1. L. A. Kelley, S. Mezulis, C. M. Yates, M. N. Wass, M. J. E. Sternberg, The Phyre2 web portal for protein modeling, prediction and analysis. *Nat Protoc* **10**, 845–858 (2015).
2. K. V. Krasileva, *et al.*, Uncovering hidden variation in polyploid wheat. *Proc National Acad Sci* **114**, E913–E921 (2017).
3. M. Van Bel, *et al.*, PLAZA 4.0: an integrative resource for functional, evolutionary and comparative plant genomics. *Nucleic Acids Res* **46**, gkx1002- (2017).
4. Q. Shi, *et al.*, Genome-wide characterization and expression analyses of the auxin/indole-3-acetic acid (Aux/IAA) gene family in barley (Hordeum vulgare L.). *Sci Rep-uk* **10**, 10242 (2020).
5. F. Tian, D.-C. Yang, Y.-Q. Meng, J. Jin, G. Gao, PlantRegMap: charting functional regulatory maps in plants. *Nucleic Acids Res* **48**, D1104–D1113 (2019).
6. U. Raudvere, *et al.*, g:Profiler: a web server for functional enrichment analysis and conversions of gene lists (2019 update). *Nucleic Acids Res* **47**, W191–W198 (2019).
7. J. T. Robinson, *et al.*, Integrative genomics viewer. *Nat Biotechnol* **29**, 24–26 (2011).
8. L. A. Kelley, S. Mezulis, C. M. Yates, M. N. Wass, M. J. E. Sternberg, The Phyre2 web portal for protein modeling, prediction and analysis. *Nat Protoc* **10**, 845–858 (2015).
9. D. Bustos-Korts, *et al.*, Exome sequences and multi-environment field trials elucidate the genetic basis of adaptation in barley. *Plant J* **99**, 1172–1191 (2019).
10. H. Ashkenazy, *et al.*, ConSurf 2016: an improved methodology to estimate and visualize evolutionary conservation in macromolecules. *Nucleic Acids Res* **44**, W344–W350 (2016).
11. A. M. Altenhoff, *et al.*, OMA orthology in 2021: website overhaul, conserved isoforms, ancestral gene order and more. *Nucleic Acids Res* **49**, gkaa1007- (2020).
12. C. Berezin, *et al.*, ConSeq: the identification of functionally and structurally important residues in protein sequences. *Bioinformatics* **20**, 1322–1324 (2004).

13. R. C. Edgar, MUSCLE: multiple sequence alignment with high accuracy and high throughput. *Nucleic Acids Res* **32**, 1792–1797 (2004).
14. M. N. Price, P. S. Dehal, A. P. Arkin, FastTree: Computing Large Minimum Evolution Trees with Profiles instead of a Distance Matrix. *Mol Biol Evol* **26**, 1641–1650 (2009).
15. I. Letunic, P. Bork, Interactive Tree Of Life (iTOL) v5: an online tool for phylogenetic tree display and annotation. *Nucleic Acids Res*, gkab301- (2021).
16. K. F. X. Mayer, *et al.*, A physical, genetic and functional sequence assembly of the barley genome. *Nature* **491**, 711–716 (2012).
17. H. Takehisa, *et al.*, Genome-wide transcriptome dissection of the rice root system: implications for developmental and physiological functions. *Plant J* **69**, 126–140 (2012).
18. Y. Sato, *et al.*, RiceXPro Version 3.0: expanding the informatics resource for rice transcriptome. *Nucleic Acids Res* **41**, D1206–D1213 (2013).
19. Y. K. Lee, *et al.*, Functionally redundant LNG3 and LNG4 genes regulate turgor-driven polar cell elongation through activation of XTH17 and XTH24. *Plant Mol Biol* **97**, 23–36 (2018).
20. J. K. Polko, *et al.*, SHOU4 Proteins Regulate Trafficking of Cellulose Synthase Complexes to the Plasma Membrane. *Curr Biol* **28**, 3174-3182.e6 (2018).
21. T. V. Humphrey, *et al.*, PERK–KIPK–KCBP signalling negatively regulates root growth in Arabidopsis thaliana. *J Exp Bot* **66**, 71–83 (2015).
22. G. K. Kirschner, *et al.*, ENHANCED GRAVITROPISM 2 encodes a STERILE ALPHA MOTIF–containing protein that controls root growth angle in barley and wheat. *P Natl Acad Sci Usa* **118**, e2101526118 (2021).
